# Celebrating the “Invisible”: The Role of Organizational Diversity Approaches on Attracting and Retaining LGBTQ + Talent

**DOI:** 10.1007/s10869-024-09975-2

**Published:** 2024-08-07

**Authors:** Kshitij Mor, Seval Gündemir, Jojanneke van der Toorn

**Affiliations:** 1 Organizational Behavior Group, Faculty of Behavioral and Social Sciences, Utrecht University, Heidelberglaan 1, 3584 CH Utrecht, Netherlands; 2 Rotterdam School of Management, Erasmus University Rotterdam, Rotterdam, Netherlands; 3 Institute of Psychology, Leiden University, Leiden, Netherlands

**Keywords:** LGBTQ +, Diversity ideology, Identity safety, Organizational attractiveness, Turnover

## Abstract

Organizations vary in diversity approaches. Some recognize and celebrate group differences (identity consciousness), while others deem these differences unimportant and deemphasize them (identity blindness). Research on diversity approaches’ impact on workplace perceptions and experiences of marginalized groups has grown but focuses mainly on “visible” groups (e.g., racial minorities, women) while largely overlooking the potential impact on “invisible” groups (e.g., LGBTQ + individuals). Integrating the diversity approaches paradigm with signaling and identity safety theory, this research addresses this oversight. Three pre-registered studies (*N*_total_ = 1318) investigate whether LGBTQ + individuals prefer identity-conscious organizations for employment because they perceive this approach as a signal of safety and acceptance for their identity. Findings reveal that identity consciousness (vs. identity blindness) is associated with increased attraction towards organizations among prospective (Studies 1 and 2) and lower turnover intentions among incumbent (Study 3) LGBTQ + workers. Identity safety (measured through employees’ sense of authenticity, belonging, and justice) mediates these relationships. Exploratory analyses indicate that, compared to their cisgender counterparts, transgender participants generally report more negative workplace experiences and expectations (Studies 1–3). Furthermore, there is suggestive evidence that transgender individuals who publicly disclose their identity may exhibit an even more positive response towards diversity-conscious organizations. We discuss the implications of these findings and conclude that identity consciousness signals and creates an identity-safe working environment for LGBTQ + individuals, improves their workplace experiences, and enhances organizations’ ability to attract and retain LGBTQ + talent.

**Supplementary Information:**

The online version contains supplementary material available at 10.1007/s10869-024-09975-2.

Despite an increasingly diversifying labor market, many organizations grapple with becoming and remaining a representation of the societies in which they operate. To hire and retain diverse talent, organizations not only have to search and recruit in different pools but also create a safe and attractive work environment for people with different backgrounds and identities. This requires effective diversity management, which pro-actively addresses the needs of (prospective) employees from minoritized groups who frequently experience struggles, distress, prejudice, and exclusion at work (Cheryan & Markus, 2020; Clair et al., 2005; Cumberbatch, 2021; Ghumman et al., 2013; van der Toorn et al., 2020; van Dijk et al., 2020).

One stream of psychology literature that is particularly relevant for creating such work environments is the diversity approaches paradigm (Apfelbaum et al., 2016; Gündemir et al., 2019; Purdie-Vaughns et al., 2008). This paradigm has outlined two types of approaches organizations can adopt. An *identity-blind* organizational approach underlines a belief that demographic differences are inconsequential and should receive minimal recognition. In this view, a focus on similarities across groups or individual-level uniqueness has a central place. An *identity-conscious* approach emphasizes instead that demographic differences should be acknowledged and celebrated (Plaut et al., 2009, 2018; Rattan & Ambady, 2013).^1^ The intergroup and interpersonal effects of these approaches have been well documented in the literature (for a recent meta-analysis, see Leslie et al., 2020). Yet, how and why these approaches contribute to organizations’ ability to attract and retain minoritized groups with relatively invisible characteristics has been missing (for an exception see, Kirby et al., 2024). Addressing this oversight in the literature, the current work examines the effects of organizations’ diversity approaches and studies whether and why these approaches may impact organizations’ ability to attract and retain a large, but relatively understudied group: lesbian, gay, bisexual, trans, or otherwise queer (LGBTQ +) employees (Rahman et al., 2020).

Integrating the diversity approaches paradigm with signaling theory (Connelly et al., 2011; White et al., 2019) and perspectives on identity safety (Kruk & Matsick, 2021; Purdie-Vaughns et al., 2008), this work advances theory in four ways. First, we offer a new critical extension of the diversity approaches paradigm to involve target groups with (partly) concealable identities. This extension is crucial for at least two reasons. Emerging research suggests that when it comes to diversity approaches, one size does not fit all. For example, research shows differential effects of diversity approaches for racial minorities and women, as well as opposing patterns of what type of approach may be most beneficial for these groups (Apfelbaum et al., 2016; Cheryan & Markus, 2020; Iyer, 2022; Martin & Phillips, 2017; Plaut et al., 2018; Purdie-Vaughns et al., 2008; Yogeeswaran & Dasgupta, 2014; for a review see, Gündemir et al., 2019). This highlights the significance of accumulating empirical knowledge on the effects of diversity approaches on various demographic groups. Such knowledge helps in understanding and reconciling diverse responses across these groups. Further, in comparison with readily visible group memberships, (partly) concealable identities present employees with additional identity management complexities (Clair et al., 2005). Compared to racial minorities and women, whose group membership is often visible to others, LGBTQ + individuals are more likely to be confronted with considerations whether to maintain an authentic and coherent sense of self at work by revealing their identity or to avoid prejudice and discrimination by concealing it (Bilimoria & Stewart, 2009; Doyle & Barreto, 2022; Griffith & Hebl, 2002; Ragins et al., 2007). A lingering theoretical question in the diversity approaches paradigm is thus how the celebration versus deemphasizing of group membership affects members of groups beyond racial minorities and women, for whom the burden of expression often lies within the individual. Further, what constitutes cues of safety and acceptance remains unclear for LGBTQ + individuals, given that previously identified safety cues such as numerical representation and role models in higher hierarchical positions (Apfelbaum et al., 2016; Johnson et al., 2019; Kruk & Matsick, 2021) may not be as straightforward due to the identities’ relative invisibility.

Second, this research contributes to theory by carefully unpacking the psychological mechanisms of *why* diversity approaches may impact organizations’ ability to attract and retain LGBTQ + talent. Drawing on signaling theory (Bird & Smith, 2005; Connelly et al., 2011; Fombrun & Shanley, 1990; White et al., 2019), we suggest that LGBTQ + employees’ responses to diversity approaches can be understood by examining the extent to which these approaches signal identity safety. Identity safety is a popular, yet surprisingly poorly understood, variable. Its measurement often includes concepts like attraction and trust (Chaney et al., 2016; Hildebrand et al., 2020; Purdie-Vaughns et al., 2008) which sometimes confounds the consequences of safety with its antecedents and processes. We resolve issues with construct contamination by clearly defining and testing the relationship between the predictors (i.e., diversity approach), processes (i.e., perceptions of key safety indicators), and outcomes (i.e., attraction, turnover intentions). Our work thus illuminates why diversity approaches affect LGBTQ + talent and enhances construct clarity within identity-safety scholarship.

Third, one limitation of existing research on attracting and retaining talent is that it primarily focused on the presence versus absence of diversity-relevant cues (Griffeth et al., 2000; Groeneveld, 2011; Lee & Zhang, 2021; McKay et al., 2007; White et al., 2019). Past studies on signaling theory have often operationalized the diversity approach as a dichotomous variable that is either present or absent in an organization without specifying the exact diversity approach. However, in the contemporary landscape, many large organizations have some form of diversity message or approach in place (Gündemir & Galinsky, 2018; Kirby et al., 2023). Thus, studying *which*, rather than *whether*, diversity approaches are present, and how they affect prospective and current employees, is a highly relevant test of central tenets of signaling theory in contemporary organizations.

Fourth, the LGBTQ + group is a broad and diverse community, consisting of several subgroups with varying degrees of stigma and unique experiences. In this study, we recognize the importance of accounting for potential within-group variability in responses. Therefore, we undertake a series of exploratory analyses to examine the response patterns as influenced by (a) individuals’ concealment versus openness about their LGBTQ + membership and (b) the role of membership in different subgroups within the LGBTQ + community.

Taken together, this research synthesizes scattered insights and advances the diversity approaches paradigm, illuminating organizations’ ability to attract and retain minoritized groups with relatively invisible characteristics. We present our theoretical model in Fig. 1.

**Fig. 1:**
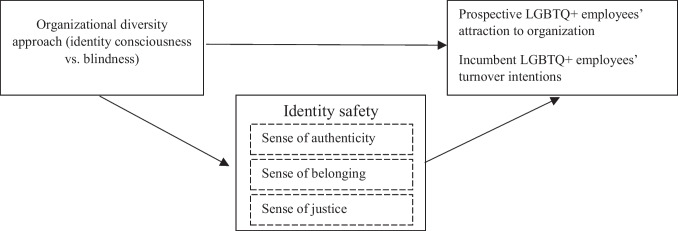
Theoretical model

## Diversity Approaches as Signals for Minoritized Employee Groups

Because of their inherently disadvantaged position, minoritized groups tend to be particularly sensitive to environmental cues communicating acceptance of one’s identity and gravitate towards organizations embodying those cues (Avery & McKay, 2006; Lee & Zhang, 2021; Lindsey et al., 2017; Thomas & Wise, 1999; van Dijk et al., 2020). The process of attending to cues to infer underlying organizational characteristics can be understood through signaling theory. According to signaling theory, there is an information asymmetry between organizations and potential employees. This asymmetry is reduced through signals that communicate competence, the type of work environment, fit, and more (Spence, 2002). A critical assumption of signaling theory is that perceivers differ in how they seek and interpret signals and show variability in their vigilance to signals (Connelly et al., 2011). That is, cues of acceptance and fairness may become especially important for groups that are most concerned about these: minoritized groups.

Most research has examined the impact of diversity approaches on racial minorities and women (Gündemir et al., 2019; Leslie & Flynn, 2022; Rattan & Ambady, 2013). For racial minorities, the benefits of an identity-conscious approach have been frequently highlighted (Leslie et al., 2020; Plaut et al., 2018). Research has, for instance, documented benefits of identity consciousness over blindness in domains ranging from improved self-esteem (Verkuyten, 2009), work engagement (Plaut et al., 2009), and sense of inclusion (Jansen et al., 2015). Notably, racial minorities’ positive responses to diversity-conscious approaches may still depend on their numeric representation at specific companies (Apfelbaum et al., 2016) or on the centrality of their racial identity (Kirby & Kaiser, 2020).

While women and racial minorities share common challenges in terms of visibility, competency concerns, pay inequality, negative stereotyping, and lack of fit concerns, research on gender and diversity approaches indicates that recommendations effective for improving the workplace experiences of racial minorities may not always yield similar benefits for women. Some research on women suggests that diversity blindness generates more favorable effects on women in the workplace, including an increased sense of agency, confidence, and pro-active behaviors (Martin & Phillips, 2017). Recent research has explained this by arguing and empirically demonstrating that because women’s workplace disadvantage is often attributed to biological or internal stereotypes, an identity-conscious approach can exacerbate these stereotypes, ultimately further disadvantaging them (Martin, 2023). However, some work nuanced these findings by showing that the benefits of identity blindness may be restricted to women with a strong career orientation, whose needs may be different from those with a family orientation (Martin et al., 2018). Other studies have hinted at a similar nuance, albeit more indirectly, by showing that approaches related to gender consciousness (such as highlighting gender-based differences) can benefit women in the workplace (Cheryan & Markus, 2020; Morgenroth & Ryan, 2018b).

As this research stream matured over the past decades, scholars increasingly call for a more tailored approach that takes critical contingencies in targeted groups’ contexts into account and make attempts to understand the mechanisms of diversity approaches shifting effects across groups. In this regard, recent research attempted to reconcile some of the prior findings on the benefits of different diversity ideologies for racial minorities and women. In a series of unique studies, Martin (2023) demonstrated that an awareness approach may be beneficial for groups whose disadvantaged position is primarily attributed to opportunity-based differences (e.g., racial minorities), whereas blindness approach may “work best” for groups with internalized (or essentialized) attributes or stereotypes (e.g., women).

## Extending the Diversity Approaches Paradigm to LGBTQ + Employees: Diversity Conscious = Identity Safe?

Because existing work on diversity approaches has focused on the study of employee groups with relatively visible identities (i.e., racial minorities and women), its utility for employee groups with concealable identities has yet to be determined. Here, we examine this paradigm in the context of a crucial target group: LGBTQ + individuals.

Sexual and gender minorities face unique challenges in organizations which differ from other identity groups widely studied within the diversity approaches paradigm. First, an LGBTQ + identity is often relatively invisible to others, which presents an information management challenge that lies within individuals. Compared to racial minorities and women, who can more easily gauge a supportive environment through numerical representation within the organizational hierarchy or by identifying role models to enhance perceived fit and signal safety (Apfelbaum et al., 2016; Banchefsky & Park, 2018; Clair et al., 2005; Johnson et al., 2019; Kruk & Matsick, 2021), LGBTQ + individuals often lack readily visible cues to assess the safety and supportiveness of their workplace. This may leave them more dependent on the diversity approach of the organization to signal information about the working environment (see Kirby et al., 2024). In addition, to find support from similar others, LGBTQ + employees have to consider whether to opt for (partial) concealment or disclosure of their identity. Both choices carry critical personal consequences that are difficult to predict in advance (Clair et al., 2005). This disclosure dilemma presents a unique source of stress for LGBTQ + individuals, which individuals with less concealable identities may not experience. Second, the expression of LGBTQ + identity has historically been subject to persecution, and it remains illegal in many parts of the world (Flores et al., 2023). Even in places where being LGBTQ + is protected by the law, LGBTQ + individuals can face stigma, harassment, and bullying because of their identity (European Union Agency for Fundamental Rights, 2013; Kuyper, 2016; Meyer, 2003). Job applicants who appear queer are perceived more negatively (Gorsuch, 2019; Granberg et al., 2020; Schilt & Westbrook, 2009; Tilcsik, 2011); LGBTQ + employees experience hiring and promotion discrimination and are often on the receiving end of everyday prejudice and microaggressions (Badgett et al., 2007; Embrick et al., 2007; McFadden & Crowley-Henry, 2016; Sears & Mallory, 2014; van Dijk et al., 2020). Considering the relatively limited attention LGBTQ + employees receive in academia and applied settings, some scholars suggest that there is a significant underestimation of antigay sentiment in the workplace (Coffman et al., 2017).

The experienced or anticipated bias among LGBTQ + workers can be understood within the framework of heteronormative ideology. This ideology prescribes a heterosexual and cis-gender identity as the standard, with defined gender roles for males and females, branding any deviations from this norm as abnormal (Cumberbatch, 2021; van der Toorn et al., 2020; Velez et al., 2021). Heteronormativity in the workplace manifests in professional standards that may conflict with LGBTQ + identity expression. The term “heteroprofessionalism” has been coined and explains how heteronormative ideals in the workplace may disadvantage LGBTQ + employees (Leslie et al., 2020; Mizzi, 2013, 2016; Salvati et al., 2021; Williams & Giuffre, 2011). It refers to the perception of “professionalism” in the workplace, which is primarily shaped by norms set by the majority, often aligning with attributes linked to masculinity, cisgender identity, and heterosexuality. This tendency can put LGBTQ + individuals, who deviate from these norms, at a disadvantage.

Heteroprofessionalism can manifest in diverse forms. Discussions involving sexuality are often marginalized in professional environments, categorized as either “irrelevant” or, in many instances, labeled as “unprofessional.” This categorization delineates sexuality as a facet of the personal sphere, distinctly separated from the professional domain. Importantly, heterosexuality escapes these negative connotations as it is often the norm and thus considered an implicit part of professional life, whereas non-heterosexual sexuality is pushed to the margins. These dynamics create an environment where conversations about sexuality are seen as relevant only for LGBTQ + individuals, reinforcing their outsider status while maintaining heterosexuality as the unspoken standard (Bizzeth & Beagan, 2023; Compton & Dougherty, 2017; Corlett et al., 2022; Cumberbatch, 2021; Mizzi, 2013, 2016; Priola et al., 2018; van der Toorn et al., 2020). Heteroprofessionalism is further reinforced by dress codes within numerous professions, and organizations often conform to traditional gender norms, emphasizing distinctions between men and women. Expressing oneself in a way that challenges these gender binaries, such as men wearing skirts or heels, is often met with disapproval and might contradict the established dress code in many workplaces (Compton & Dougherty, 2017; Lehtonen, 2016; Resnick & Galupo, 2019; Schilt & Westbrook, 2009).

Taken together, the separation of LGBTQ + identities from the work sphere may lead to hesitancy in assessing LGBTQ + workplace needs, and reporting LGBTQ + inclusion in diversity reports, thereby eliminating safety cues and sources of information about the degree of genuine diversity commitment of the organization (Klarenaar et al., 2022; Wilton et al., 2020). Further, the myriad of potential negative consequences associated with being openly queer at work, norms associated with sexual and gender identity disclosure, and the relatively invisible nature of an LGBTQ + identity may motivate employees to conceal their identity (Griffith & Hebl, 2002; King et al., 2017; Thuillier et al., 2021).

Against this backdrop, compared to other demographic groups, employees with LGBTQ + identities have less access to visible cues within organizations to infer how their group membership is viewed. For example, racial minorities and women can often infer diversity signals not only from institutional support signals but also from representational cues (e.g., role models or numeric representation; Apfelbaum et al., 2016; Clair et al., 2005; Johnson et al., 2019; Kruk & Matsick, 2021; Wilton et al., 2020). For LGBTQ + individuals, signals of institutional support thus become especially important to shape their views of an organization and its openness to their identity groups (Johnson et al., 2019; Kirby et al., 2024; Kruk & Matsick, 2021; Thuillier et al., 2021).

Here, we examine a potential effect of these cues as signals for “identity safety.” While identity safety has been recognized as a key mechanism for why minoritized groups respond to diversity approaches (Kirby & Kaiser, 2020; 2024; Purdie-Vaughns et al., 2008), there is great variability in how the term “identity safety” is conceptualized and measured in the literature (Johnson et al., 2019; Kruk & Matsick, 2021; Pietri et al., 2018). Measures assessing identity safety often include elements such as trust, organizational attractiveness, and work commitment (Chaney et al., 2016; Hildebrand et al., 2020; Purdie-Vaughns et al., 2008), leading, at times, to a blurred distinction between identity safety, its outcomes, and the underlying processes. In response, we reviewed the identity safety literature and observed that, despite the absence of a standardized measure or definition, there were recurring aspects in the way the construct of identity safety is conceptualized. The common denominator across studies is that identity safety involves an experience or expectation that one’s identity is (a) welcomed and valued and (b) will form no hindrance in a specific context (Chaney et al., 2016; Walton et al., 2015).

The experience of being welcomed and valued involves a workplace that fosters the expression of individual identity and encourages meaningful connections with colleagues. This underscores the significance of both a sense of authenticity and belonging for identity safety. Identity safety cannot be accomplished without a sense of belonging, as it would mean the criterion of being welcomed is not satisfied. However, a sole emphasis on belonging, without considering authenticity, might compel individuals to conform to group dynamics, risking the compromise of crucial aspects of their identity in the workplace (Jansen et al., 2014; Shore et al., 2011, 2018).

In addition to the freedom to express oneself and build positive connections with others, identity safety also involves an absence of devaluation or bias because of one’s identity. This facet differs from the other two as the focus of evaluation shifts from oneself (e.g., Do I feel a sense of belonging?) to the organization (e.g., Does the organization employ equitable processes?). In this context, identity safety pertains to the extent to which individuals perceive the organizational environment as a potential source of inequitable treatment or fair procedures based on their group membership (Ambrose & Schminke, 2009; Colquitt, 2001; Colquitt et al., 2013). This facet is best captured by assessing the extent to which target groups see the organization as a fair entity.

In sum, to experience identity safety is to exist in an environment where one has a sense that their identity is embraced and respected and where the organization adheres to principles of justice. Thus, identity safety is characterized by a sense of authenticity, belonging, and fair treatment.

We argue that LGBTQ + individuals will be more attracted to organizations with an identity-conscious approach compared to an identity-blind approach because this will signal a more identity safe environment to them (Chaney et al., 2016; Howansky et al., 2021; Purdie-Vaughns et al., 2008). Considering the stigma attached to an LGBTQ + identity, active acknowledgement, celebration, and incorporation of an LGBTQ + identity in the diversity approach of an organization (as is the case for identity-conscious approaches) will better facilitate feelings of authenticity and belonging (Hatzenbuehler, 2009; Hebl et al., 2014; Köllen, 2013). Furthermore, given the pervasiveness of heteronormativity at work, a focus on similarity, as emphasized in the identity-blind approach, may cue concerns about heteroprofessionalism and the threat of prejudice for LGBTQ + individuals (Cumberbatch, 2021; Mizzi, 2013, 2016; van der Toorn et al., 2020). Hence, despite the intention of a blind approach to promote equality and fairness, LGTBQ + individuals may in fact perceive it as less fair. Past and emergent research supports this view by showing that signals communicating an explicit recognition and celebration of gender and sexual identity can indeed prompt identity safety among LGBTQ + individuals. For example, research in the USA shows that observing others’ use of personal pronouns signals procedural fairness and fosters positive organizational attitudes among gender and sexual minorities (Johnson et al., 2021). Other work demonstrates that organizational cues for diversity consciousness, such as the existence of LGBTQ + supportive policies or diversity statements, can reduce anxiety and encourage a sense of belonging and identity disclosure among LGBTQ + individuals (Griffith & Hebl, 2002; Kirby et al., 2024). The link with disclosure is critical because in environments where LGBTQ + employees feel comfortable disclosing their identity, they perform better, report feelings of inclusion and identification with the organization, and experience less work-related stress and negative affect (Clair et al., 2005; Hebl et al., 2014; Martinez et al., 2017; Webster et al., 2018). Thus, LGBTQ + employees are likely to use cues of identity consciousness to infer a work environment that offers identify safety (Griffith & Hebl, 2002; Joo et al., 2016; Kahn et al., 2015; King et al., 2017; Kirby et al., 2024; Purdie-Vaughns et al., 2008).

## Attraction and Retention of LGBTQ + Employees

Based on the above-presented integration of signaling theory and the diversity approaches paradigm, we have proposed that LGBTQ + individuals make identity-safety inferences based on organizational diversity approaches. We argue that the benefits of identity consciousness will extend to organizations’ ability to promote diversity and inclusion.

One area where this becomes evident is how attractive an organization appears to LGBTQ + talent. As prospective employees (e.g., job seekers) often face constraints in accessing accurate information about an organization and its attributes, they rely on signals to reduce their uncertainty (Connelly et al., 2011). That is, prospective employees infer unknown information from available cues (Lindsey et al., 2017). Research demonstrates that organizational cues that convey a safe working environment and signal fairness and trust appear more attractive to job applicants (Capell et al., 2018; Joo et al., 2016; Kahn et al., 2015; Leung et al., 2021). Studies specifically focusing on underrepresented groups consistently show that dimensions associated with identity safety, which we argue will be triggered by a conscious approach, can critically drive these groups’ attraction to organizations. For example, when signals during the recruitment process indicate anticipated belonging to women, their intentions to apply increase (Hentschel et al., 2021). Conversely, cues for reduced anticipated belonging (Georgeac & Rattan, 2023) or devaluation (Puncheva-Michelotti et al., 2024) diminish women’s reports of organizational attraction. Moreover, diversity signals communicating inclusive and bias-free environments can boost racial minority workers’ attraction towards organizations (for reviews, see Avery & McKay, 2006; McKay, 2024). One recent study unraveled that sexual and gender minorities may exhibit similar responses to such cues and inferences. Specifically, inferences of a positive diversity climate based on organizational cues have been shown to heighten both organizational attraction and person-organization fit among members of this group (Bradley et al., 2023).

Taken together, the literature suggests that an identity-conscious organization will signal an environment that meets the identity safety needs of LGBTQ + employees, thereby enhancing their attraction to these organizations. In other words, we anticipate that LGBTQ + individuals will perceive companies that prioritize identity consciousness as more appealing work environments (than those emphasizing blindness) due to the perceived higher level of identity safety they signal:

- *H1: LGBTQ* + *individuals will find identity-conscious organizations to be more attractive than identity-blind organizations.*
- *H2: The relationship between organizational diversity ideology and organizational attractiveness will be mediated by the anticipated identity safety (i.e., anticipated authenticity, belonging, and justice).*

Another critical domain for the relationships of interest pertains to organizations’ ability to retain LGBTQ + talent. While signaling theory has been primarily utilized to understand outsiders’ responses to organizations (Connelly et al., 2011), its relevance may extend beyond these responses and explain events within organizations (Lindsey et al., 2017). To test the utility of signaling in explaining incumbents’ responses, we examine whether a conscious approach signals identity safety to incumbent LGBTQ + employees, enhancing organizations’ ability to retain this group of employees. This extension is critical for two reasons. First, research shows that LGBTQ + employees experience more distress, harassment, and exclusion compared to their cis-hetero counterparts (Bilimoria & Stewart, 2009; Embrick et al., 2007; Galupo & Resnick, 2016; McFadden & Crowley-Henry, 2016). These unfavorable experiences create a push factor and put them at risk for higher rates of turnover (Deery et al., 2011; Griffeth et al., 2000). Second, limiting turnover (especially among minoritized employee groups) is crucial for companies given the financial and reputational costs associated with it.

Prior research underscores the critical role of employees’ perceptions of the work environment in their retention. Organizations’ ability to communicate key components of identity safety, such as a sense of belonging and justice, is linked to employees’ intentions to remain with the company (Choi, 2011; Das & Baruah, 2013). Diversity management research offers evidence that such cues may also substantially benefit the retention of minoritized employees. For example, favorable diversity climates, evaluated in broad terms (i.e., whether antidiscrimination policies exist, if organizations offer equal access to training or publicize their diversity principles; Groeneveld, 2011; McKay et al., 2007; Wagner, 2017), can be helpful for retaining minoritized groups. Notably, some studies have shown that key subcomponents of identity safety may drive these beneficial effects. For example, when organizational diversity-related cues signal fairness to employees of color, they report lower turnover intentions and higher work engagement (Buttner et al., 2010; Lee et al., 2021). Similarly, a sense of belonging, a central component of identity safety, is a critical correlate of intentions to stay in work domains where minorities are underrepresented (Rainey et al., 2018). A sense of inclusion, encompassing both belonging and authenticity subcomponents, is strongly related to workplace satisfaction among minoritized groups, a variable linked to their likelihood of staying employed at a company (Jansen et al., 2015).

In sum, based on the literature, we expect an identity-conscious organization to evoke a sense of identity safety among incumbent workers (Howansky et al., 2021; Sabharwal et al., 2019), making them less likely to want to leave the organization. Specifically, we hypothesize:

- *H3: Perceptions of organizational identity consciousness will be negatively associated with turnover intentions among LGBTQ* + *employees.*
- *H4: The relationship between organizational identity consciousness and turnover intentions will be mediated by perceived identity safety (i.e., perceived authenticity, belonging, and justice).*

## Exploring the Role of Identity Concealment and Intragroup Variation

Given the emphasis placed on the importance of concealment versus disclosure for LGBTQ + well-being at work (Hatzenbuehler, 2009; Hebl et al., 2014; Köllen, 2013), our study aims to investigate the potential impact of LGBTQ + employees’ level of identity disclosure or openness on their perception of diversity cues. Specifically, we explore whether the effect of the diversity approach differs for individuals who are more versus less open about their identity. LGBTQ + individuals, who tend to be more open about their queerness, often consider this aspect of their identity as more central to their self-concept, shaping their worldview (Suppes et al., 2021). Research also indicates that when an identity is central, individuals become more attuned to potential threats and stigma associated with that identity (Hinton et al., 2022). Consequently, LGBTQ + individuals who are more open about their identity, compared to those who are less open, may be more sensitive to signals of safety or potential discrimination due to their heightened awareness of prejudice and discrimination. As a result, they may be inclined to seek environments that explicitly signal safety and acceptance of their identity, such as identity-conscious organizations.

Furthermore, it is important to acknowledge that the LGBTQ + community is not a homogenous one, and different subgroups within the community may have diverse experiences and different responses to diversity cues. The experiences of individuals with only a minoritized sexual orientation can differ from those with a minoritized gender identity and from those with both. Previous research has highlighted that among LGBTQ + individuals, transgender individuals, especially those who also belong to a sexual minority group, face some of the most challenging outcomes (Cech & Rothwell, 2020; McFadden & Crowley-Henry, 2016; Pepper & Lorah, 2008). Notably, even within diversity and inclusion policies for LGBTQ + inclusivity, the specific concerns and experiences of transgender individuals are understudied (Lehtonen, 2016; Ozturk & Tatli, 2016). Moreover, individuals with a minoritized gender identity, such as transgender individuals, often encounter greater difficulty concealing their identity compared to those with only a minoritized sexual orientation. Physical transformations resulting from hormone treatments, conforming attire to match one’s gender identity, or changing one’s name can inadvertently “out” transgender individuals, placing them at heightened risk of facing adverse reactions as they may be perceived as challenging the traditional norms of a workplace (Brewster et al., 2014; Diamond et al., 2011; Granberg et al., 2020; Hennekam & Ladge, 2023; Mizzi, 2016). Considering the additional challenges faced by transgender individuals and their heightened visibility within the LGBTQ + community, we anticipate that they may experience comparatively worse outcomes across various domains than cis-gendered individuals. Consequently, we explore whether transgender individuals’ gravitation towards identity-conscious organizations that explicitly communicate safety and acceptance is more pronounced than that of cis-gender individuals. This addition is important in light of scholarly calls to better highlight the unique experiences of transgender individuals (Cancela et al., 2024; Law et al., 2011; McFadden & Crowley-Henry, 2016; Pepper & Lorah, 2008; Sangganjanavanich & Headley, 2013). In an effort to bridge this gap, we aim to conduct comparative exploratory analyses, distinguishing between transgender and cisgender participants, to address and highlight the distinct experience of this understudied group.

## Overview of Studies

We examined our hypotheses across three pre-registered studies. Studies 1 and 2 were vignette experiments, and Study 3 used a survey design. The studies were built in Qualtrics and distributed using the crowdsourcing platform Prolific. Participants were prescreened, and only individuals who lived in the UK and who identified as lesbian, gay, bisexual, transgender, or otherwise queer (LGBTQ +) were invited through the platform. To ensure representation, the samples consisted of at least 25% transgender participants. Information about participants’ age, work status, gender identity, and sexual orientation was collected in each study. Text field entries for the gender identity and sexuality questions (“I prefer to self-describe”) were manually scanned and recategorized when necessary. Participants who identified as heterosexual and cis-gender, and participants with missing data were removed prior to analyses. We supplemented our collected data with the ethnicity data collected by Prolific to explore the potential intersectional implications of our results. Unless stated otherwise, all scales were measured using a seven-point Likert scale (1 = *Strongly disagree*, 7 = *Strongly agree*). At the end of each study, participants were debriefed and had an opportunity to provide comments about the study.

The sampling plan, data exclusions (if any), all experimental manipulations, and measures are described in the main text, the Supplementary Online Materials (SOM), or can be found on the Open Science Framework. All tables and figures are included in the manuscript or can be found in the SOM. All analyses were conducted using IBM SPSS 28 and Rstudio, and ethics approval was obtained for all studies (21–467, 22–0334, 22–0456). The preregistration details, data, materials, and codes are available on the Open Science Framework (https://osf.io/ysx2w/?view_only=a4805c46090e40af966a376ee3fde562).

## Study 1

This study tested whether LGBTQ + individuals will, on average, find an identity-conscious organization to be more attractive than an identity-blind organization (H1).

### Participants and Procedure

An a-priori power analysis indicated that we needed a sample size of 398 participants (*d* = 0.25, 1-*β* = 0.80, *α* = 0.05; Faul et al., 2007). We oversampled to account for possible missing data and requested 420 responses. We received 407 complete responses (*M*_age_ = 31.63, *SD*_age_ = 10.73; for demographics see Table 1, for means and correlations see Table 2).^2^

**Table 1:** Demographic composition of participant samples of Studies 1–3

Demographic characteristics	Study 1		Study 2		Study 3	
	*n*	%	*n*	%	*n*	%
Work status						
Employed full-time	188	46.2	217	47	333	74.8
Employed part-time	71	17.4	90	19.5	112	25.2
Unemployed looking for work	27	6.6	22	4.8	-	-
Unemployed not looking for work	43	10.6	46	10	-	-
Retired	7	1.7	6	1.3	-	-
Student	71	17.4	81	17.5	-	-
Gender group						
Male	104	25.6	151	32.7	141	31.7
Female	265	65.1	234	50.6	237	53.3
Non-binary	29	7.1	60	13	51	11.5
Genderfluid	5	1.2	8	1.7	6	1.3
Agender	0	0	4	0.8	4	0.9
Self-described	4	1.0	5	1.1	6	1.3
Transgender identity						
Yes	57	14	112	24.2	102	22.9
No	350	86	349	75.5	343	77.1
Other	0	0	1	0.2	0	0
Sexual orientation						
Gay	58	14.3	66	14.3	70	15.7
Lesbian	60	14.7	46	10	80	18
Bisexual	204	50.1	212	45.9	195	43.8
Queer	24	5.9	36	7.8	26	5.8
Asexual	34	8.4	40	8.7	25	5.6
Pansexual	26	6.4	58	12.6	42	9.4
Heterosexual	1	0.2	4	0.9	5	1.1
Self-described	0	0	0	0	2	0.4
Ethnicity						
White	363	89.2	416	90	398	89.4
Black	4	1	9	1.9	7	1.6
Asian	13	3.2	15	3.2	15	3.4
Mixed	19	4.7	18	3.9	19	4.3
Other	1	.2	2	.4	3	0.7
Missing/unknown	7	1.7	2	.4	3	0.7

**Table 2:** Descriptive statistics and correlations of Studies 1–3

Variable	*M (SD)*	1	2	3	4	5
*Study 1*						
1. Age	31.63 (10.73)	-				
2. Organizational attractiveness	4.56 (1.67)	.08	-			
3. Workplace openness about gender identity	6.03 (1.85)	.14^**^	.04	-		
4 Generalized openness about gender identity	6.19 (1.50)	.13^**^	.03	.97^**^	-	
5. Workplace openness about sexual orientation	4.03 (2.28)	.17^**^	− .02	.24^**^	.21^**^	-
6. Generalized openness about sexual orientation	4.59 (1.86)	.12^**^	.00	.21^**^	.20^**^	.94^**^
*Study 2*						
1. Age	30.48 (10.02)	-				
2. Organizational attractiveness	4.76 (1.53)	.12^*^	-			
3. Anticipated authenticity	4.78 (1.89)	.04	.83^**^	-		
4. Anticipated belonging	4.94 (1.55)	.05	.84^**^	.81^**^	-	
5. Anticipated justice	4.97 (1.33)	.09^+^	.78^**^	.82^**^	.81^**^	
*Study 3*						
1. Age	32.73 (9.41)	-				
2. Organizational diversity approach	4.87 (1.37)	.02	-			
3. Turnover intentions	3.71 (2.04)	− .16^**^	− .39^**^	-		
4. Perceived authenticity	5.12 (1.60)	.12^*^	.55^**^	− .54^**^	-	
5. Perceived belonging	4.79 (1.61)	.15^**^	.54^**^	− .58^**^	.79^**^	-
6. Perceived justice	5.09 (1.46)	.13^**^	.44^**^	− .58^**^	.71^**^	.76^**^

*N*_Study 1_ = 407(403 for gender identity parameters due to missing data points); *N*_Study 2_ = 462; *N*_Study 3_ = 445. ^+^*p* < .10, ^*^p < .05, ^**^p < .01 s

Participants were asked to form an impression of an organization based on limited information. After providing informed consent, participants were randomly assigned to either an identity blind or an identity conscious condition and rated that organization’s attractiveness. Finally, participants completed demographic questions.

### Materials and Measures

Depending on the condition, participants were presented with a version of the webpage of a fictitious organization named Wynn Inc., including its diversity mission statement (texts are modeled after Purdie-Vaughns and colleagues, 2008). The mission statement in the identity-blind condition emphasized ignoring differences and fostering equality through a focus on similarities. A sample phrase is “While other firms mistakenly focus on their staff’s diversity, we at Wynn Inc. train our workforce to embrace their similarities.” In the identity-conscious condition, the mission statement emphasized the value of diversity and embracing differences and included phrases such as “While other firms mistakenly try to shape their staff into a single mold, we at Wynn Inc. believe that embracing our differences enriches our culture.”

The perceived attractiveness of the organization was assessed using the five-item attractiveness subscale of the organizational attraction scale (Highhouse et al., 2003). A sample item is “For me, this company would be a good place to work.” (*α* = 0.95). To test whether the manipulation was successful, participants rated the extent to which they thought Wynn Inc. valued differences (Kirby & Kaiser, 2020; Purdie-Vaughns et al., 2008)*.*

To measure participants’ openness, they rated how open they were about their gender identity and sexual orientation on a 7-point scale, in the following domains: “To colleagues,” “To supervisor/management,” “To family,” “To friends,” and “In general.” This was adapted from the outness scale of Mohr and Fassinger (2000), with the wording of the questions changed to not only measure outness but also openness about sexual orientation and gender identity. Participants reported their age and employment status and indicated their gender identity and sexual orientation (see Morgenroth & Ryan, 2018a). Data about participants’ ethnicity were retrieved from Prolific and merged with the data collected through our survey.

### Results

#### Manipulation Check

Participants in the identity conscious condition more strongly perceived the organization to value group differences in the work setting (*M* = 6.14, *SD* = 1.12) than participants in the identity-blind condition (*M* = 2.67, *SD* = 1.83, *t*(349.48 =  − 23.33, Cohen’s *d* = 1.52, *p* < 0.001, 95% CI [− 3.77, − 3.18]). The manipulation was thus successful.

#### Hypothesis Testing

Consistent with H1, participants found an identity-conscious organization to be more attractive (*M* = 5.38, *SD* = 1.31) than an identity-blind organization (*M* = 3.80, *SD* = 1.62, *t*(396.01) =  − 10.89, *p* < 0.001, 95% CI [− 1.87, − 1.30], Cohen’s *d* = 1.48).

#### Exploratory Analyses: The Role of Openness and Intragroup Variation

We first investigated if participants’ attraction to organizations embodying different diversity approaches varied based on how open they were about their gender identity and/or sexual orientation both at work and in general. Organizational diversity approach was dummy coded, with identity-blind ideology (coded as 0) as the reference condition. To determine how open participants were at work, we derived *workplace openness* by calculating a mean score of openness “To colleagues” and openness “To supervisor/management.” We derived *generalized openness* by calculating a mean score of all openness items.^3^

**Fig. 2:**
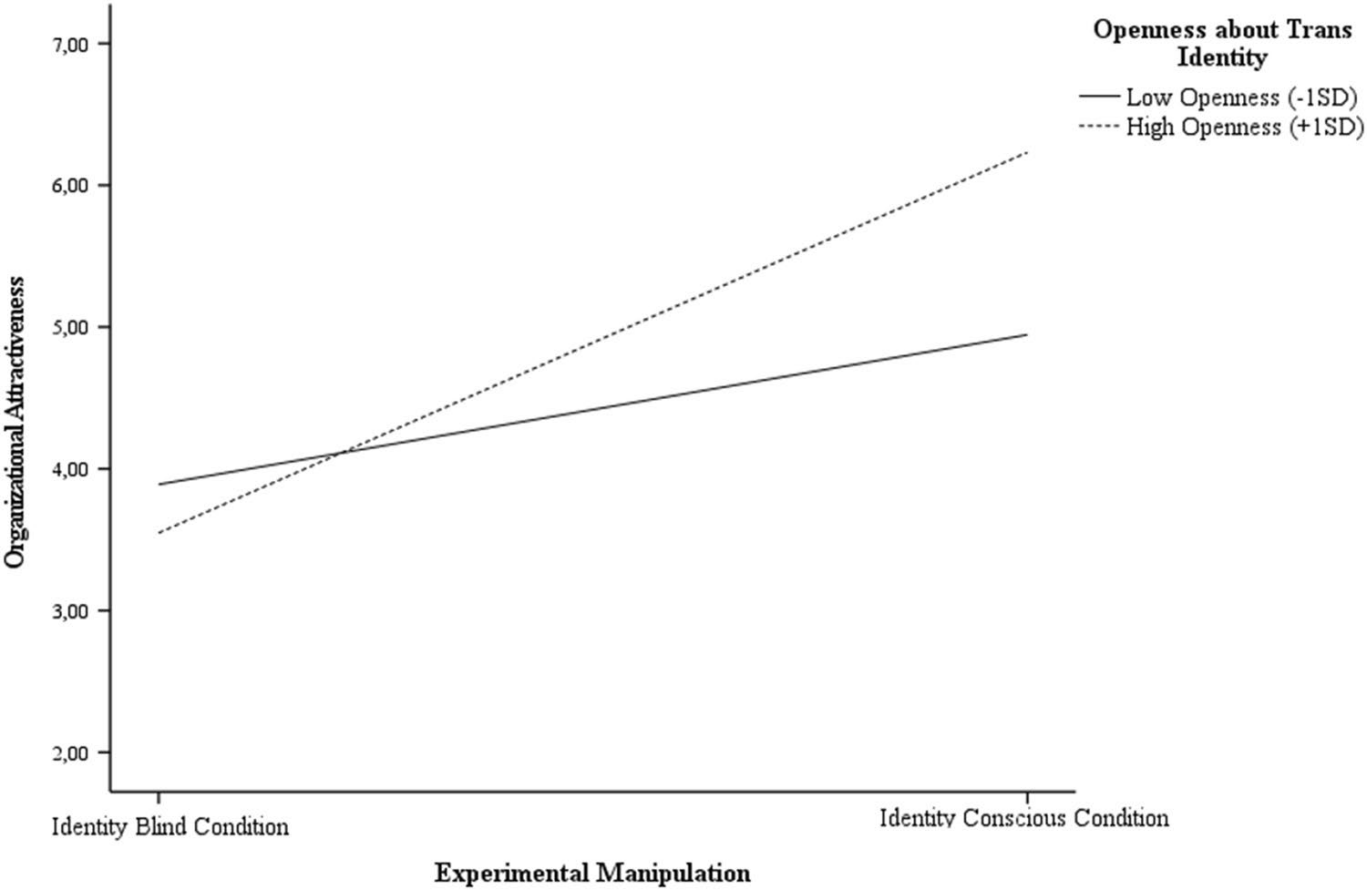
Moderation effect of generalized openness about gender identity on organizational attractiveness

We performed a series of regression analyses on organizational attractiveness with an organizational diversity approach, openness, and their interaction as our IVs. We conducted separate analyses, including openness about gender identity and openness about sexual orientation. Openness was centered at the mean. Among sexual minority participants (*n* = 406; i.e., almost the full sample), we obtained no significant interactions between diversity approach and either type of openness about sexual orientation (*p*s > 0.224; see Table 3). Among transgender participants (*n* = 57), we observed no significant interaction between diversity approach and workplace openness about gender identity (*p* = 0.090; see Table 3), but we did obtain a significant interaction between diversity approach and generalized openness about gender identity, revealing that transgender participants who were generally more open about their gender identity found the identity conscious organization to be more attractive (*b*_interaction_ = 0.46, *SE* = 0.20, *p* = 0.023, 95% CI [0.07, 0.86]; see Fig. 2 for a visualization).

**Table 3:** Multiple regression analyses to assess effects of diversity approach and openness about gender identity and sexual orientation on organizational attractiveness in Study 1

	Model 1 – intercept only			Model 2 – openness variation			Model 3 – interaction effects		
	*B(SE)*	95% CI	*p*	*B(SE)*	95% CI	*p*	*B(SE)*	95% CI	*p*
Workplace openness about gender identity									
Diversity approach	1.92 (0.35)	1.21, 2.63	< .001	1.88 (0.36)	1.16, 2.59	< .001	1.86 (0.35)	1.16, 2.56	< .001
Openness				0.09 (0.08)	− 0.07, 0.25	.282	− 0.03 (0.10)	− 0.24, 0.18	.770
Diversity approach × openness							0.27 (0.16)	− 0.04, 0.59	.090
Workplace openness about sexual orientation									
Diversity approach	1.58 (0.15)	1.29, 1.87	< .001	1.58 (0.15)	1.29, 1.87	< .001	1.58 (0.15)	1.29, 1.87	< .001
Openness				− 0.001 (0.03)	− .06, .06	.984	− 0.04 (0.05)	− 0.13, 0.05	.393
Diversity approach × openness							0.08 (0.07)	− .0.05, 0.21	.224
Generalized openness about gender identity									
Diversity approach	1.92 (0.35)	1.21, 2.63	< .001	1.89 (0.36)	1.18, 2.60	< .001	1.87 (0.34)	1.18, 2.56	< .001
Openness				0.10 (0.10)	− 0.11, 0.30	.342	− 0.10 (0.13)	− 0.35, 0.16	.449
Diversity approach × openness							0.46 (0.20)	0.07, 0.86	.023
Generalized openness about sexual orientation									
Diversity approach	1.58 (0.15)	1.29, 1.87	< .001	1.58 (0.15)	1.29, 1.87	< .001	1.58 (0.15)	1.29, 1.87	< .001
Openness				0.01 (0.04)	− 0.07, 0.09	.777	− 0.02 (0.05)	− 0.12, 0.09	.770
Diversity approach × openness							0.06 (0.08)	− 0.10, 0.22	.441

Diversity approach is coded 1 = identity consciousness and 0 = identity blindness. Analyses including openness about gender identity were only conducted for transgender participants (*n* = 57). Analyses including openness about sexual orientation were conducted with the full sample (*n* = 407)

Next, we examined potential differences between transgender and cisgender individuals among our participants. We performed a regression analysis to explore (a) possible differences between cisgender (coded as 0) and transgender (coded as 1) participants in organizational attraction (i.e., a main effect of gender identity) and (b) a possible interaction effect between organizational diversity approach and gender identity. We found no significant main or interaction effects (*ps* > 0.369; see Table 4).

**Table 4:** Multiple regression analyses to assess effects of diversity approach and gender identity on organizational attractiveness in Study 1

	Model 1 – intercept only			Model 2 – intragroup variation			Model 3 – interaction effects		
	*B(SE)*	95% CI	*p*	*B(SE)*	95% CI	*p*	*B(SE)*	95% CI	*p*
Variables									
Diversity approach	1.59 (0.15)	1.30, 1.87	< .001	1.59 (0.15)	1.30, 1.88	< .001	1.53 (0.16)	1.22, 1.85	< .001
Gender identity				0.09 (0.21)	− 0.32, 0.51	.660	− 0.08 (0.28)	− 0.64, 0.48	.784
Diversity approach × gender identity							0.38 (0.43)	− 0.45, 1.22	.369

Diversity approach is coded 1 = identity consciousness and 0 = identity blindness; gender identity is coded 1 = transgender and 0 = cisgender

#### Exploratory Analyses: The Role of Race/Ethnicity

We conducted an additional linear regression analysis to explore (a) potential differences between white (coded a 0) and non-white (coded as 1) participants in organizational attraction and (b) a possible interaction effect between race/ethnicity and organizational approach. We found no significant main or interactive effects (*ps* > 0.239).

### Discussion

In support of H1, this study provided evidence that organizations that recognize, value, and celebrate group-based diversity may be more effective at attracting LGBTQ + talent than organizations ignoring those differences.

Exploratory analyses suggest that while this preference for identity-conscious organizations is not contingent on how open LGBTQ + individuals are about their sexual orientation, some subgroup differences may occur. Specifically, transgender participants who were generally more open about their gender identity found identity-conscious organizations to be more attractive. Given that gender identity for transgender individuals may not always conform to the gender binary, concealing their gender identity and expression can be challenging (Cumberbatch, 2021; Law et al., 2011; McFadden & Crowley-Henry, 2016; Morgenroth & Ryan, 2021). Consequently, it is plausible that those who are more open about their gender identity may be drawn to organizations that explicitly include and embrace diverse social identities in their diversity approach. The pattern of results suggests an intriguing possibility that the degree of openness about gender identity among transgender participants may influence their perception of diversity cues. However, it is important to interpret these findings with caution due to the exploratory nature of the analyses and the limitations posed by the relatively small sample size of the subgroup.

Considering the majority of null findings regarding the variable of openness in Study 1, we decided not to pursue further exploration of this variable in our subsequent studies. Since an error with the sampling tool resulted in a limited representation of transgender participants in Study 1, we took extra precautions in the next studies to ensure we have adequate transgender representation in our sample.

## Study 2

Study 2 sought to replicate and expand upon the results from Study 1. In addition to re-testing the direct relationship between ideology and organizational attractiveness (H1), the goal was to uncover whether identity safety perceptions could be the psychological mechanism underlying LGBTQ + individuals’ preference for an identity-conscious organization over an identity-blind one (H2).

### Participants and Procedure

Based on sample norms from other studies that used similar mediation models and a supplementary Monte Carlo analysis which suggested a sample of 400–500 to achieve power values around 0.8 (Ambrose & Schminke, 2009; Joo et al., 2016; Leung et al., 2021), we requested 500 responses on Prolific. We received 499 responses. Thirty-seven participants did not meet our pre-registered criteria and were excluded before analysis. Our final sample included 462 participants (*M*_age_ = 30.48, *SD*_age_ = 10.02, for demographics, see Table 1, for means and correlations, see Table 2).

The procedure was largely identical to Study 1. One difference was that participants additionally indicated their anticipated experiences of authenticity, belonging, and justice at the company.

### Materials and Measures

The diversity approach vignettes, the organizational attractiveness measure (*α* = 0.95), the manipulation check, and demographics measures were identical to the ones used in Study 1.

Anticipated authenticity was measured using four items from an adapted version of the authenticity subscale from the Perceived Group Inclusion Scale (PGIS; Jansen et al., 2014). A sample item is “This organization will allow me to present myself the way I am.” (*α* = 0.99).

Anticipated belonging was measured using four items from an adapted version of the belongingness subscale from the PGIS (Jansen et al., 2014). A sample item is “This organization will give me the feeling that I belong.” (*α* = 0.96).

Anticipated justice was measured using four items from an adapted version of the Perceived Overall Justice Scale (POJ; Ambrose & Schminke, 2009). A sample item is “Overall, I will be treated fairly by this organization.” (*α* = 0.95).^4^

### Results

#### Measurement and Manipulation Checks

We conducted a series of confirmatory factor analyses (CFAs) on the authenticity, belonging, and justice items to check whether the slight rewording had affected the factor structure of the instruments (not pre-registered). For authenticity and belonging, all items loaded highly (> 0.80) on the latent variable for each instrument, respectively. For the justice scale, all items, apart from one item (“Most of the people who work here will say they are often treated unfairly.”), loaded acceptably on the latent variable (> 0.75). Since removing this item did not change the results, we kept it in analyses as originally planned.

Participants in the identity conscious condition more strongly perceived the organization to value group differences in the work setting (*M* = 6.14, *SD* = 1.16) than participants in the identity-blind condition (*M* = 2.80, *SD* = 1.78), *t*(401.73) =  − 23.99, *p* < 0.001, 95% CI [− 3.62, − 3.07], Cohen’s* d* = 1.50), indicating our manipulation was successful.

#### Hypothesis Testing

Consistent with H1, participants in the identity conscious condition found the organization more attractive (*M* = 5.40, *SD* = 1.16) than participants in the identity blind condition (*M* = 4.14, *SD* = 1.59, *t*(426.37) =  − 9.77, *p* < 0.001, 95% CI [− 1.52, − 1.01], Cohen’s* d* = 1.40).

**Fig. 3:**
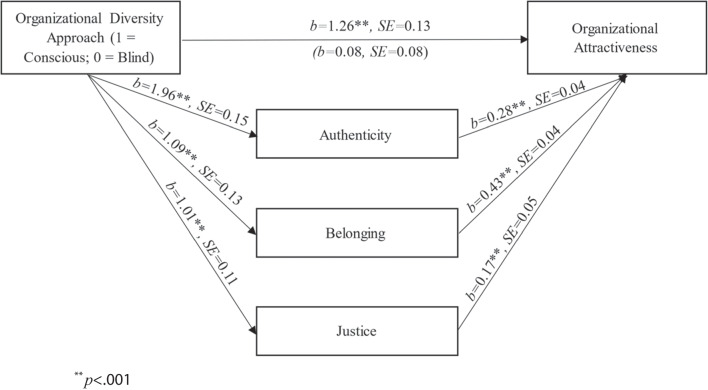
Path coefficients for the indirect effects in Study 2. Note: *n* = 462; ^**^p < .001

We tested our full model (Fig. 1) using the PROCESS procedure in SPSS (Model 4; Hayes, 2017). We estimated the indirect effects of the diversity approach on organizational attractiveness through anticipated authenticity, belonging, and justice.^5^Organizational diversity approach was dummy coded, with identity-blind ideology (coded as 0) as the reference condition. To test the indirect effects in the model, we calculated a bias-corrected confidence interval for each indirect effect based on 10,000 bootstrap samples. All path coefficients can be found in Fig. 3.

Organizational identity consciousness (vs. blindness) predicted higher levels of anticipated authenticity, belonging, and justice. All three identity safety indicators were positively associated with attractiveness. There was a significant indirect effect of organizational diversity ideology on organizational attractiveness through authenticity (*b*_indirect_ = 0.55, *SE* = 0.12, 95% CI [0.34, 0.80]), belonging (*b*_indirect_ = 0.47, *SE* = 0.10, 95% CI [0.28, 0.68]), and justice (*b*_indirect_ = 0.17, *SE* = 0.07, 95% CI [0.05, 0.31]).

#### Exploratory Analyses: Intragroup Variation

We performed a series of regression analyses to explore (a) possible differences between cisgender (coded as 0) and transgender (coded as 1) participants in their reported organizational attraction and anticipated authenticity, belonging, and justice, and (b) a possible interaction effect between organizational approach and gender identity.

Across conditions, transgender participants reported significantly lower organizational attractiveness (*b* =  − 0.50, *SE* = 0.15, *p* = 0.001 95% CI [− 0.79, − 0.20]), anticipated belonging (*b* =  − 0.52, *SE* = 0.16, *p* < 0.001, 95% CI [− 0.83, − 0.22]), and anticipated justice (*b* =  − 0.33, *SE* = 0.13, *p* = 0.013, 95% CI [− 0.59, − 0.07]) than cisgender participants. The difference in anticipated authenticity between transgender and cisgender participants was not significant (*p* = 0.128). We found no support for an interaction between organizational approach and gender identity on the dependent measures (*p*s > 200; see Table 5).

**Table 5:** Multiple regression analyses of intragroup variation and interaction effect of transgender identity on dependent variables in Study 2

	Model 1 – intercept only			Model 2 – intragroup variation			Model 3 – interaction effects		
	*B(SE)*	95% CI	*p*	*B(SE)*	95% CI	*p*	*B(SE)*	95% CI	*p*
Organizational attractiveness									
Diversity approach	1.26 (0.13)	1.00, 1.52	< .001	1.26 (0.13)	1.01, 1.52	< .001	1.17 (0.15)	0.88, 1.46	< .001
Gender identity				− 0.50 (0.15)	− 0.79, − 0.20	.001	− 0.69 (0.21)	− 1.10, − 0.27	.001
Diversity approach × gender identity							0.39 (0.30)	− 0.20, 0.98	.200
Anticipated authenticity									
Diversity approach	1.96 (0.15)	1.66, 2.25	< .001	1.96 (0.15)	1.66, 2.26	< .001	1.94 (0.17)	1.60, 2.28	< .001
Gender identity				− 0.27 (0.18)	− 0.62, 0.08	.128	− 0.30 (0.25)	− 0.79, 0.19	.225
Diversity approach × gender identity							0.07 (0.35)	− 0.63, 0.76	.848
Anticipated belonging									
Diversity approach	1.09 (0.14)	0.82, 1.35	< .001	1.09 (0.13)	0.83, 1.35	< .001	1.04 (0.15)	0.74, 1.34	< .001
Gender identity				− 0.52 (0.16)	− 0.83, − 0.22	< .001	− 0.63 (0.22)	− 1.06, − 0.19	.005
Diversity approach × gender identity							0.21 (0.31)	− 0.40, 0.82	.502
Anticipated justice									
Diversity approach	1.01 (0.11)	0.78, 1.23	< .001	1.01 (0.11)	0.79, 1.23	< .001	1.03 (0.13)	0.78, 1.29	< .001
Gender identity				− 0.33 (0.13)	− 0.59, − 0.07	.013	− 0.28 (0.19)	− 0.65, 0.09	.131
Diversity approach × gender identity							− 0.10 (0.27)	− 0.62, 0.43	.718

Diversity approach is coded 1 = identity consciousness and 0 = identity blindness; gender identity is coded 1 = transgender and 0 = cisgender

#### Exploratory Analyses: The Role of Race/Ethnicity

We conducted a series of linear regression analyses to examine the difference between white (coded a 0) vs non-white (coded as 1) participants on organizational attraction, anticipated authenticity, belonging, and justice. We also tested for an interaction effect between organizational approach and race/ethnicity. We found no significant main or interactive effects on any of the dependent measures (*ps* > 0.188).

#### Robustness Check

We noticed that some participants from Study 1 also took part in Study 2. To adjust for potential inflated effects due to the sample overlap, we re-ran the results of Study 2 after removing all participants that also participated in Study 1 (adjusted *n* = 396). The results and conclusions were identical.

### Discussion

Replicating Study 1, Study 2 showed that LGBTQ + individuals perceive an identity-conscious organization as more appealing for employment than an identity-blind organization. This preference is explained by identity safety. LGBTQ + individuals believe that in an identity-conscious company, they can be more authentic, belong more, and be treated fairly.

Exploratory analyses demonstrated that transgender individuals report lower attraction, belonging, and justice than cisgender individuals, regardless of the organization’s diversity approach. This finding aligns with research on transgender individuals in the labor market, which consistently highlights the heightened challenges faced by this group. (Law et al., 2011; McFadden & Crowley-Henry, 2016; Pepper & Lorah, 2008).

## Study 3

The first two studies showed that employing a diversity-conscious approach can help organizations attract LGBTQ + talent. In Study 3, we test whether the benefits of a diversity-conscious approach extend to incumbents, reducing LGBTQ + employees’ turnover intentions (Cohen et al., 2016; Griffeth et al., 2000). We further gauge the role of the three indicators of identity safety in explaining the relationship between perceived diversity approach and turnover intentions.

Understanding whether a conscious diversity approach predicts employees’ workplace withdrawal behaviors has two key implications. First, it broadens our understanding of the reach of diversity approaches beyond experimental settings. This complements the first two studies in important ways and adds to the ecological validity of our theoretical model. Second, attracting talent is only one part of the diversity puzzle; many organizations have difficulty retaining talent from traditionally underrepresented groups who have entered their company. Indeed, research shows that minority employees show higher turnover than majority employees (Deery et al., 2011; Hofhuis et al., 2014; Jones & Harter, 2005). Given that turnover is highly costly for organizations, understanding the conditions under which this may be limited is of great importance for organizations.

### Participants and Procedure

Based on similar a-priori considerations as in Study 2, we requested 500 responses on Prolific. Fifty-five participants did not meet our criteria and were excluded. Our final sample included 445 participants (*M*_age_ = 32.73, *SD*_age_ = 9.41; for demographics, see Table 1; for means and correlations, see Table 2).

In the survey description, participants read that we were interested in their experiences at work. After providing informed consent, participants completed a short questionnaire about their demographics. They then reported the diversity approach of their current organization, turnover intentions, sense of authenticity, belonging, and justice at work.

### Materials and Measures

Organizational diversity approach was measured with nine items: four items assessing the perceived identity consciousness of own organization and five items assessing perceived identity blindness, adapted from Dang and colleagues (2022). Example items include “My organization behaves in ways that ignore employees’ demographic background” and “My organization believes that employees’ demographic differences should be acknowledged and valued.” We conducted a principal components analysis, using a direct oblimin rotation (Conway & Huffcutt, 2003). The results showed two factors with eigenvalues of 4.61 (four diversity consciousness items and two reverse-coded blindness items accounting for 51.25% of the variance) and 1.48 (three remaining items accounting for 16.46% of the variance). Consistent with our pre-registered approach and other studies providing theoretical support and empirical precedent for approaching ideology as a unitary construct (see Koenig & Richeson, 2010; Martin & Phillips, 2017), we created a single measure of diversity approach using the items for the first factor with higher scores representing a more conscious approach and lower scores representing a more blind approach (*α* = 0.90). We ran a series of robustness checks on our model and findings (e.g., controlling for a scale comprising the three other items), which yielded no meaningful changes in our result patterns or interpretations. These can be found in the SOM.

The measures for demographic variables, authenticity (*α* = 0.98), belonging* (α* = 0.96), and justice (*α* = 0.96) were the same as in Study 2. Turnover intentions were measured using two items from Lawler and colleagues (1975). The items were “I often think about quitting” and “I will probably look for a new job in the next year” (*r* = 0.73).^6^

### Results

#### Initial Check: Adjusting for Common Method *Bias* (CMB)

To mitigate potential risks associated with common method bias (CMB)—artificially inflated relationships between variables (Spector & Brannick, 2010)—we followed the scholarly recommendations (Simmering et al., 2015) and included a marker variable that was theoretically unrelated to any of our other variables (i.e., preference for the color green). Controlling for the effects of this variable had no notable effects on our findings.

#### Hypothesis Testing

**Fig. 4:**
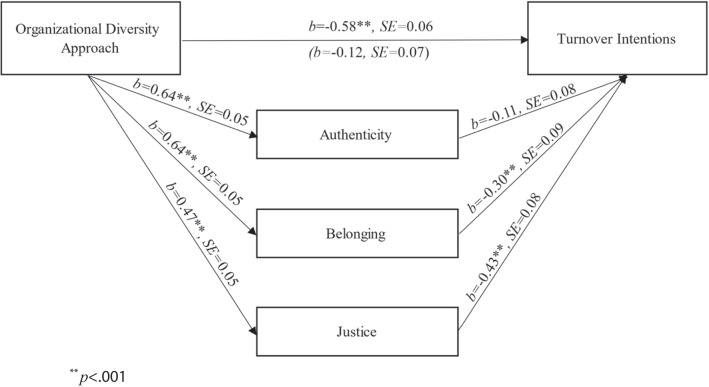
Path coefficients for the indirect effects in Study 3. Note: *n* = 445; ^**^p < .001

A linear regression supported H3: higher levels of perceived organizational identity consciousness were related to lower turnover intentions among LGBTQ + employees (*b* =  − 0.58, *SE* = 0.07, *p* < 0.001, 95% CI [− 0.71, − 0.46]). We then tested the theoretical model (see Fig. 1) using the PROCESS procedure in SPSS (Model 4; Hayes, 2017), employing a similar strategy as in Study 2. One difference was the predictor: perceived organizational diversity approach was a continuous variable, with higher scores indicating a more conscious organization (see Fig. 4 for all path coefficients). The results showed that greater perceived identity consciousness predicted a higher sense of authenticity, belonging, and justice. Belonging and justice, but not authenticity, were in turn associated with lower turnover intentions. There were significant indirect effects of the organizational diversity approach on turnover intentions through belonging (*b*_indirect_ =  − 0.19, *SE* = 0.06, 95% CI [− 0.31, − 0.07]) and justice (*b*_indirect_ =  − 0.20, *SE* = 0.05, 95% CI [− 0.30, − 0.11]). The indirect effect through authenticity (*b*_indirect_ =  − 0.07, *SE* = 0.05, 95% CI [− 0.18, 0.04]) was not significant.

#### Exploratory Analyses: Intragroup Variation

Using a series of linear regression analyses, we estimated how transgender participants (coded as 1), compared to cisgender participants (coded as 0), experienced turnover intentions, belonging, authenticity, and justice. We also tested for an interaction effect between organizational diversity ideology and transgender identity.

Across conditions, transgender (vs. cisgender) participants reported significantly higher turnover intentions (*b* = 0.66, *SE* = 0.21, *p* = 0.002, 95% CI [0.25, 1.08]) and lower authenticity (*b* =  − 0.53, *SE* = 0.15, *p* < 0.001, 95% CI [− 0.82, − 0.23]), belonging (*b* =  − 0.60, *SE* = 0.15, *p* < 0.001, 95% CI [− 0.90, − 0.30]), and justice (*b* =  − 0.37, *SE* = 0.15, *p* = 0.014, 95% CI [− 0.66, − 0.07]).

Of all our measures, we found a single significant interaction for sense of belonging (*p* = 0.049). However, we refrain from drawing definitive conclusions regarding this interaction due to the large number of tests conducted, the relatively large *p*-value, and the absence of similar findings in any of our other studies. The details of the regression slopes can be found in Table 6.

**Table 6:** Multiple regression analyses of intragroup variation and interaction effect of transgender identity on dependent variables in Study 3

	Model 1 – intercept only			Model 2 – intragroup variation			Model 3 – interaction effects		
	*B(SE)*	95% CI	*p*	*B(SE)*	95% CI	*p*	*B(SE)*	95% CI	*p*
Turnover intentions									
Diversity approach	− 0.58 (0.07)	− 0.71, − 0.46	< .001	− 0.55 (0.07)	− 0.68, − 0.43	< .001	− 0.52 (0.08)	− 0.67, − 0.37	< .001
Gender identity				0.66 (0.21)	0.25, 1.08	.002	1.27 (0.71)	− 0.13, 2.67	.074
Diversity approach × gender identity							− 0.13 (0.15)	− 0.42, 0.16	.369
Authenticity									
Diversity approach	0.64 (0.05)	0.55, 0.73	< .001	0.62 (0.05)	0.52, 0.71	< .001	0.60 (0.05)	0.49, 0.70	< .001
Gender identity				− 0.53 (0.15)	− 0.82, − 0.23	< .001	− 0.86 (0.50)	− 1.85, 0.13	.088
Diversity approach × gender identity							0.07 (0.10)	− 0.13, 0.28	.488
Belonging									
Diversity approach	0.64 (0.05)	0.54, 0.73	< .001	0.61 (0.05)	0.52, 0.70	< .001	0.55 (0.06)	0.45, 0.66	< .001
Gender identity				− 0.60 (0.15)	− 0.90, − 0.30	< .001	− 1.56 (0.51)	− 2.56, − 0.57	.002
Diversity approach × gender identity							0.21 (0.11)	0.00, 0.41	.049
Justice									
Diversity approach	0.47 (0.05)	0.38, 0.56	< .001	0.45 (0.05)	0.36, 0.54	< .001	0.41 (0.05)	0.31, 0.52	< .001
Gender identity				− 0.37 (0.15)	− 0.66, − 0.07	.014	− 1.01 (0.50)	− 1.99, − 0.03	.043
Diversity approach × gender identity							0.14 (0.10)	− 0.06, 0.34	.176

Diversity approach is a continuous variable with higher scores indicating a more conscious organizations. Gender identity is coded 1 = transgender and 0 = cisgender

#### Exploratory Analyses: The Role of Race/Ethnicity

We conducted a series of linear regression analyses to examine the difference between white (coded a 0) vs non-white (coded as 1) participants on turnover intentions, authenticity, belonging, and justice. We also tested for an interaction effect between organizational diversity approach and race/ethnicity. We found no main or interactive effects of race/ethnicity on turnover intentions, authenticity, belonging, or justice (*ps* > 0.106).

#### Exploratory Analyses: Employee Tenure

Employees’ work experience could potentially have an impact on their sensitivity to diversity signals or influence the weight they might place on diversity signals. For example, employees who have worked at the company longer may have a deeper understanding of the importance of an identity-conscious versus identity-blind approach to the organizational climate compared to those who have worked for the company for a shorter period. To investigate this possibility, we used a series of linear regression analyses examining the interaction between participants’ tenure (the number of years worked at their current organization) and the organization’s diversity approach. We examined how this interaction influenced their turnover intentions, as well as their sense of authenticity, belonging, and justice within the organization.

We found a negative relationship between tenure and turnover intentions (*b* =  − 0.04, *SE* = 0.02, *p* = 0.041, 95% CI [− 0.07, − 0.00]); the longer participants had worked at their organization, the lower their turnover intentions were. Tenure was not associated with a sense of authenticity, belonging, or justice. Intriguingly, we observed tenure by organizational diversity approach interactions, on turnover intentions (*b* = 0.03, *SE* = 0.01, *p* = 0.040, 95% CI [0.00, 0.05]), sense of authenticity (*b* =  − 0.03, *SE* = 0.01, *p* = 0.009, 95% CI [− 0.04, − 0.01]), and belonging (*b* =  − 0.02, *SE* = 0.01, *p* = 0.033, 95% CI [− 0.04, − 0.00]). Unpacking these interactions showed that while diversity consciousness benefits all employees, their benefits are more pronounced among LGBT + employees with shorter a shorter tenure (see Figs. 1S, 2S, and 3S in the SOM).

### Discussion

Study 3 offered additional evidence to the utility of a diversity-conscious approach for organizations aiming to enhance and retain LGBTQ + representation. Adding to the insights from the first two studies that demonstrated that identity consciousness can pull new LGBTQ + employees to companies, this study showed that this approach can also help companies hold on to the LGBTQ + talent they already have.

Additional analyses revealed intriguing insights. First, consistent with Study 2 and past research, transgender employees’ workplace experiences appeared to be less positive than those of cisgender employees (Law et al., 2011; McFadden & Crowley-Henry, 2016; Pepper & Lorah, 2008). Further, while all LGBTQ + employees responded positively to diversity consciousness, we did not find strong support for the additional benefits of diversity consciousness for transgender employees. Second, the benefits of diversity consciousness for LGBTQ + employees are more pronounced among those with relatively shorter tenures. This aligns with the idea that new employees may rely more on the signals conveyed by an organization’s diversity approach compared to those with longer tenures, who may have additional or alternative sources of information to form their impressions.

## General Discussion

Three pre-registered studies extended the diversity approaches paradigm to understand how organizations can attract and retain LGBTQ + talent. We predicted and found that LGBTQ + individuals find identity conscious organizations to be more attractive employers than identity-blind organizations and that this preference is explained by anticipated authenticity, belonging, and justice (identity safety). Critically, we also demonstrated that the benefits of identity consciousness extend to incumbents: LGBTQ + employees report lower turnover intentions to the extent that they perceive their organization to be identity conscious, primarily because these contexts improve their sense of belonging and perceived justice.

### Theoretical Implications

This work advances the diversity approaches paradigm by extending it to the workplace experiences of minoritized employees with group memberships that are often not readily visible to others. A clear show of support through identity-conscious messaging appears to have a positive effect on LGBTQ + individuals, who may find it more difficult to ascertain a safe working environment due to the lack of availability of visible cues of safety that are available to minorities with more visible stigma (Apfelbaum et al., 2016; Banchefsky & Park, 2018; Clair et al., 2005; Johnson et al., 2019; Kruk & Matsick, 2021). The current contribution is among the first to examine diversity approaches for LGBTQ + workers, along with the recent work by Kirby and colleagues’ (2024), which found a positive impact of identity consciousness on LGBTQ + workers’ identity disclosure, perceived fairness, and belonging. Our study constructively extends these findings to the broader domain of organizational attractiveness and employee turnover and further expands upon identity safety as a mechanism underlying these relationships.

Our contribution to the broader discussions within the diversity approach paradigm lies in elucidating whether LGBTQ + individuals prefer and benefit from identity-conscious approaches, akin to racial minorities, or from identity-blind approaches, as observed with women in previous research. Our consistent finding that LGBTQ + employees respond more positively to a conscious approach fits prior empirical findings regarding racial minorities. One possible explanation of this overlap is that both racial minorities and LGBTQ + individuals face disadvantage in the workplace that is attributed to social inequities and opportunity-based differences; thus, a conscious approach can effectively place emphasis on these inequities and highlight a need and support for inclusive policies. In contrast, the disadvantage faced by women in the workplace is often attributed to perceived biological and internal differences. Accordingly, research has demonstrated that a focus on consciousness accentuates biological stereotypes, in ways that limit women’s potential for success (Martin, 2023).

It is intriguing to observe that, despite research suggesting that a blind approach may better cater to the needs of women in the workplace (Gündemir et al., 2019; Leslie et al., 2020; Martin & Phillips, 2017), LGBTQ + women in our samples predominantly express a preference for a conscious approach. For instance, across studies, we find no interactive effects of participant gender, suggesting that women’s responses do not systematically differ from men’s or other genders’ (see SOM for details). We posit that, akin to the phenomenon of ethnic prominence, queer women in this study may be prioritizing the more stigmatized aspect of their identity—their queer identity—over their identity as women. Consequently, they appear to gravitate towards a conscious organizational approach, which proves more beneficial for their queerness, as opposed to a blind organizational approach that might be more advantageous for them as women (Levin et al., 2002; Taylor et al., 2012).

Given the relatively concealable nature of an LGBTQ + identity, we sought to understand whether the effects of an identity-conscious organization would differ across individuals with differing degrees of openness about their identity. Potential shifts in the benefits of identity consciousness across persons who are more or less “out” would have different theoretical implications. For example, if effects are restricted to individuals who are out, this would suggest their reach is limited to public interactions. However, if their impact extends to individuals who are “closeted,” this could imply a broader impact on internal psychological responses. Our lack of findings with regards to one’s openness around their sexual orientation, in conjunction with the consistently documented advantages of identity consciousness for LGBTQ + individuals in various studies, suggests that even for closeted LGBTQ + individuals—whose needs may be challenging to assess—employing an overarching conscious diversity approach can be beneficial. In addition to the sexual identity, we also considered the role of one’s openness about their gender identity. Outside of one instance where we observed that transgender individuals who were overall more open about their gender identity showed a stronger preference for conscious organizations, we found no strong evidence for a moderating role of gender identity openness. Overall, these findings suggest that identity consciousness has far-reaching benefits for LGBTQ + employees, even when they can and/or choose to conceal their identity.

Our work offers valuable peripheral insights on the workplace experiences of transgender individuals, a subgroup often overlooked in research. The additional finding that transgender individuals overall anticipate and experience lower authenticity, belonging, and justice and show higher turnover intentions (even in more identity-conscious organizations) underscores the heightened struggles this subgroup encounters compared to their cisgender counterparts (Cancela et al., 2024; Martinez et al., 2017; McFadden, 2015; McFadden & Crowley-Henry, 2016). Given that nearly all transgender participants in our study also did not identify as heterosexual, this additional finding contributes to the intersectionality literature, suggesting an additive effect of multiple stigmatized gender and sexual identities on reduced positive experiences and expectations in the workplace (Berdahl & Moore, 2006). For future research, we would recommend active recruitment of heterosexual transgender participants to enable a nuanced examination of the distinctive effects arising from being both a sexual and gender minority, contrasting with the experiences of those who solely identify as a gender minority. Although our exploratory findings do not indicate an additional advantage of identity consciousness for transgender employees compared to cisgender employees, the positive responses among transgender employees to conscious approaches (as opposed to blind approaches) suggest that organizations implementing a conscious approach may be better equipped to provide a sense of safety and create an appealing workplace for this subgroup of LGBTQ + individuals.

This research examines the psychological mechanisms triggered by diversity approaches and highlights that LGBTQ + (prospective) employees’ positive responses to a conscious approach are explained by experiences of identity safety. Former work has been ambiguous in conceptualizing identity safety, often confounding its predictors, experiences, and consequences. By disentangling critical dimensions of identity safety experiences—authenticity, belonging, and justice—this work offers additional construct clarity to the identity safety literature. That is, identity safety is distinct from its antecedents (e.g., organizational cues such as diversity messages) or its consequences (e.g., employee withdrawal behaviors) and should be carefully defined and operationalized in empirical work.

The discrepancy between findings regarding anticipated (Studies 1 and 2) and experienced (Study 3) authenticity suggests that the expectation of authenticity and belonging may be more distinguishable than their actual experience. Our results demonstrate that while identity consciousness activates all three safety dimensions, their impact on LGBTQ + employee responses unfolds in different ways and depends on whether one is a prospective versus an incumbent employee. Moreover, our findings suggest that, while much research construes and studies authenticity and belonging in conjunction under the umbrella of inclusion (e.g., Jansen et al., 2014), perceivers may differentiate between them depending on the context. That is, scholars should consider the theoretical disentanglement of these constructs as separately meaningful to employees.

### Practical Implications

A key practical implication of this research is that organizations that want to create a work environment that attracts LGBTQ + talent and reduces turnover should consider incorporating identity consciousness in their diversity approach. They should explicitly communicate this approach in job ads and internal company communication with employees. In addition, we find that use of an identity-conscious approach targets multiple aspects of the diversity puzzle, in that it is not only useful in attracting LGBTQ + talent but also plays a role in retaining said talent.

Additionally, we find that the impact of the diversity approach on turnover intentions, authenticity, and a sense of belonging is more pronounced among newer LGBTQ + employees compared to their longer-tenured counterparts. We posit that this discrepancy arises because LGBTQ + employees may gradually rely less on cues derived from an organization’s diversity approach over time. For recent LGBTQ + hires, the diversity approach may serve as one of the primary signals of a safe working environment. Longer-tenured employees can identify similar individuals within the organization and discern additional cues of safety beyond the diversity approach. This underscores the importance of the HR communication of organizational diversity approaches as part of onboarding activities. As new employees, particularly those from traditionally underrepresented groups like LGBTQ + individuals, join the company, they may be particularly attentive to signals of identity consciousness. Organizations that embrace a conscious approach and proactively communicate their stance stand to benefit by fostering a more inclusive environment and facilitating talent retention.

### Limitations and Future Directions

Our study was not without limitations. The primary focus on LGBTQ + identity led us to forego recruiting a more ethnically diverse sample, limiting our ability to explore potential intersectional dynamics related to the impact of diversity approaches and safety signaling on racially minoritized individuals. While we were able to conduct exploratory analyses using meta-data from the data collection platform, which resulted in null effects, we are unable to draw strong conclusions from these given that only ~ 10% of our sample in each study represented non-white individuals. The lack of focus on intersectional dynamics is a common problem in the diversity management literature. Individuals are often categorized into a single minoritized identity, and the effects of other aspects of their identity and how they interact with diversity approaches are left unattended (Kruk & Matsick, 2021; Martin & Phillips, 2017; Purdie-Vaughns & Walton, 2011). While the current work is unique in unveiling the LGBTQ + responses to diversity approaches, future work should delve deeper into the complexities of these responses by explicitly exploring the interaction with race/ethnicity.

Further, the first two studies examined the effects of diversity approaches in a fictitious setting. While ecological validity concerns are offset by Study 3, future research should study LGBTQ + candidates’ job application behavior as a factor of organizational diversity approach in more realistic settings.

Another avenue for future research could involve broadening the scope of impact assessments related to diversity approaches by incorporating additional dependent measures. Although our current study primarily focused on metrics aligned with organizational objectives, such as talent attraction and retention, diversity approaches might yield broader benefits for the well-being and satisfaction of the target group (see Kirby et al., 2024, for empirical support for such benefits in the context of identity disclosure). Expanding our understanding of such impacts would extend beyond a strictly organization-centric perspective in management science. Furthermore, our research scrutinized the influence of diversity approaches through vision statements—abstract ideals rather than concrete policies. Considering previous findings indicating that the abstraction level of a pro-diversity focus yields different effects (see Bradley et al., 2023; Yogeeswaran & Dasgupta, 2014), investigating the impact of specific diversity-focused policies could offer valuable complementary insights. In this regard and also in light of the complexities highlighted in this study, scholarly work should explicitly unpack the unique ways organizations can signal identity consciousness to (prospective) LGBTQ + employees.

## Conclusion

While research on the diversity approaches paradigm has expanded, its utility for employee groups with invisible social identities remained largely unknown. The current work demonstrates that organizations that embrace and communicate an identity conscious approach will attract and retain more LGBTQ + talent.

## Supplementary Information

Below is the link to the electronic supplementary material.

Supplementary file1 (DOCX 105 kb)

## Data Availability

The processed and anonymized datasets analyzed during the current study are available in the Open Science Framework repository https://osf.io/ysx2w/?view_only=a4805c46090e40af966a376ee3fde562.

## Footnotes

1 The specific approaches to diversity in a local setting can take various forms and include different components that reflect these two overarching approaches. For example, an identity-blind organization can embrace an assimilationist, meritocratic, and/or individual uniqueness-focused form. These specifications are beyond the scope of this work and have been discussed in detail in prior work (Gündemir et al., 2019). Here, we adopt the prevailing operationalizations of the two overarching approaches commonly employed in organizations.

2 Due to an error with Prolific, we were unable to recruit the intended number of transgender participants for the first study; the analyses with transgender participants are still presented but should be interpreted with caution given the small sample size.

3 We included a measure of generalized openness because participants evaluate a fictitious organization in this study. While their responses to the workplace openness measure are likely influenced by factors specific to their current workplace, the generalized openness measure may reflect a dispositional tendency. Consequently, we deemed it relevant to take their generalized openness into account in their assessment of the organization depicted in the vignette.

4 We additionally measured and explored the role of personal diversity ideology in Study 2. Further details can be found in the SOM.

5 Given that these three aspects are subcomponents of an overarching safety variable, one might wonder whether they should be analyzed as a single construct rather than as separate parts. We chose to analyze the three paths separately because it provides more detailed information about potential variations in strength. Furthermore, a Confirmatory Factor Analysis (CFA) has demonstrated that a three-factor model fits the data significantly better than a one-factor model. Details of the CFA can be found in the SOM.

6 We additionally measured and explored the role of supervisor diversity approach in Study 3. Further details can be found in the SOM.
