# Supplementary material for: Celebrating the “Invisible”: The Role of Organizational Diversity Approaches on Attracting and Retaining LGBTQ + Talent
Source: J Bus Psychol. 2024 Aug 7;40(3):593–617. doi: 10.1007/s10869-024-09975-2 (PMC12049394; doi:10.1007/s10869-024-09975-2)
Supplement: Supplementary file 1 — Supplementary file1 (DOCX 105 kb) [file 10869_2024_9975_MOESM1_ESM.docx]

**Online Supplement**

Study 1: Additional Analyses

Study 2: Additional Methodological Details and Analyses

Study 3: Additional Methodological Details and Analyses

Means Tables per Gender Identity and Sexual Orientation for All Studies

Correlation Table for Final Organizational Diversity Approach Scale from Study 3

Graphs of Interaction Effect Between Diversity Approach and Tenure on Turnover, Authenticity, and Belonging in Study 3

**Study 1**

**Results – Additional Analyses**

***Interaction between Identity Consciousness, Transgender Identity, and Openness***

We conducted an additional analysis to examine a potential three-way interaction between organizational diversity approach, gender identity (i.e., cis-gender vs. transgender identity), and openness regarding one's gender identity. This analysis sought to further explore and complement the observed interaction effect between openness about gender identity and organizational diversity approach (*p*=.023) for transgender (but not cis-gender) participants. The three-way interaction was not significant.

***Moderation by Gender***

We conducted an additional linear regression analysis to explore (a) potential differences between female (coded a 1) and non-female (coded as 0) participants in organizational attraction, and (b) a possible interaction effect between female identity and organizational approach. We found no significant main or interactive effects.

**Study 2**

**Methods - Additional Material**

***Personal Diversity Ideology***

The personal diversity ideology of the participants was measured using an adapted version of the gender blindness (α=.66) and gender awareness scales (α=.78) from Hahn and colleagues (2015). Each scale consists of four items. Example items include: “The differences between different demographic groups should be acknowledged and celebrated.” and “All humans are fundamentally the same, regardless of their demographic group.” (1 = *Strongly Disagree*, 7 = *Strongly Agree*).^[[1]](#footnote-1)^

**Results**

***Confirmatory Factor Analysis of Identity Safety Construct***

To examine the factor structure of our identity safety construct we conducted a CFA and tested a three-factor model (with authenticity, belonging, and justice as separate factors) and a one-factor model. We further conducted an ANOVA to compare the three-factor model with the one-factor model. We found that the three factor model (*AIC*=14987, *BIC*=15115, *χ2* =349.84) had a significantly better fit (*p*<.001) compared to a single factor model (*AIC*=17143, *BIC*=17259, *χ2* =2512.36).

***Moderation by Gender***

We conducted an additional linear regression analysis to explore (a) potential differences between female (coded a 1) and non-female (coded as 0) participants in organizational attraction, anticipated authenticity, belonging and justice, and (b) a possible interaction effect between female identity and organizational approach. We found no significant main or interactive effects.

***Comparing Indirect Effects***

We compared the indirect effects of the mediators from our main model in Study 2. The pairwise contrast between authenticity and justice (*b_contrast_*=0.38, *SE*=0.14, 95%CI [0.13, 0.66]), and belonging and justice (*b_contrast_*=0.30, *SE*=0.14, 95%CI [0.03, 0.57]) were also significant. The pairwise contrast between authenticity and belonging was not significant (*b_contrast_*=-0.08, *SE*=0.18, 95%CI [-0.44, 0.27]).

***Personal Diversity Ideology***

We re-ran the main mediation model from Study 2 with the mean scores on both personal diversity ideology scales (identity blind and identity conscious) added as covariates. Including personal diversity ideology as a control did not change any of our results or intepretations.

**Study 3**

**Methods - Additional Material**

***Tenure***

Participants reported the number of years they had been working at their current place of work. Due to the nature of responses (e.g., including ‘years’ or ‘half’), this variable had to be manually corrected and transformed into a numerical variable in SPSS.

***Marker Variable***

To mitigate potential common method bias, we included a marker variable that was theoretically unrelated to our variables of interest. Participants were asked to indicate their preference for the color green. Preference was measured using three items and was adapted from Simmering and colleagues (2015). Example items include: “I like the color green” and “I prefer green to other colors.” (1 = *Strongly Disagree*, 7 = *Strongly Agree;* α=.86).

***Organizational Diversity Knowledge***

On a seven-point Likert-scale, participants rated the extent to which they have a good sense of the diversity approach of their organization (1 = *Strongly Disagree*, 7 = *Strongly Agree*).

***Supervisor Diversity Knowledge***

On a seven-point Likert scale, participants rated the extent to which they have a good sense of the diversity approach of their supervisor (1 = *Strongly Disagree*, 7 = *Strongly Agree*).

***Supervisor Diversity Approach***

Supervisor diversity approach was measured with nine items: four items assessing perceived identity consciousness of the direct supervisor and five items assessing perceived identity blindness. Participants were asked to think about their direct supervisor or the one they interact with most if they had multiple (Dang et al., 2022). Example items include: “My supervisor behaves in ways that ignore employees’ demographic background.” and “My supervisor believes that employees’ demographic differences should be acknowledged and valued.” (1 = *Strongly Disagree*, 7 = *Strongly Agree*).

This scale was almost identical to the organizational diversity approach scale. We conducted a principal components factor analysis, using a direct oblimin rotation (Conway & Huffcutt, 2003). The results showed two factors with eigen values 4.61 (four diversity consciousness items and three reverse coded blindness items accounting for 51.25% of the variance) and 1.48 (two blindness items accounting for 16.46% of the variance).

Supervisor diversity approach had one additional item that loaded on the first factor as compared to organizational diversity approach. For consistency we created a unitary construct using the same items as the ones we used for organizational diversity approach (α=.91).

**Results**

***Robustness Checks***

To ensure the robustness of our results from the mediation model in Study 3, we re-ran the Hayes Process Macro (Model 4) multiple times with the following changes and found no difference in the result patterns:

1. A single mean of all organizational diversity approach items was used as predictor.
2. The mean of the second factor we found in the EFA of the organizational diversity approach scale was added as a covariate.
3. The mean of the marker variable was added as a covariate.
4. The mean of the marker variable was added as a mediator – the marker variable was not a significant mediator.
5. Participants’ knowledge about the diversity approach of the organization was added as a covariate.
6. The number of years participants had worked at the organization (i.e. tenure) was added as a covariate.
7. Mean of supervisor diversity approach was added as a covariate.
8. A subset of individuals who were knowledgeable about the diversity approach of their organization (i.e., those who scored 5 or higher on the item assessing knowledge about organizational diversity approach) were used as our data sample.

Additionally, we ran a regression with organizational diversity approach mean and marker variable mean as predictors of turnover intentions. The marker variable did not significantly predict turnover intentions, and adding the marker variable did not significantly influence the effects of organizational diversity approach on turnover intentions.

***Comparing Indirect Effects***

We compared the indirect effects of the mediators from our main model in Study 3. None of the pairwise contrasts were found to be significant.

***Additional Analyses with Perceived Supervisor Diversity Approach***

Considering that employees often work closely with their supervisors and may thus be especially sensitive and affected by the diversity approach endorsed by their supervisors (Dang et al., 2022), we explored the role of perceived supervisor diversity approach in our theoretical model.

First, we ran a linear regression model to examine whether supervisor diversity approach predicted turnover intentions. Consistent with our results from organizational diversity approach we found that higher levels of perceived supervisor identity consciousness were related to lower turnover intentions amongst LGBTQ+ employees (*b*=-0.50, *SE*=0.07, *p*<.001, 95% CI [-0.63, -0.36]).

We then ran a linear regression model to check whether the effects of supervisor diversity approach on turnover intentions still held when organizational diversity approach was also present in the model. Both perceived supervisor consciousness and organizational consciousness were significantly related to lower turnover intentions amongst LGBTQ+ employees (*b_supervisor_*=-0.18, *SE_supervisor_*=0.09, *p*=.043, 95% CI [-0.36, -0.01]; *b_organization_*=-0.47, *SE_organization_*=0.09, *p*<.001, 95% CI [-0.64, -0.29]).

We ran a final regression model and included an interaction term between supervisor and supervisor diversity approach. The interaction term did not significantly relate to turnover intentions.

We furthermore wanted to explore the predictive role of supervisor diversity approach in our mediation model. We tested our full model using the PROCESS procedure in SPSS (Model 4; Hayes, 2017). We estimated the indirect effects of supervisor diversity approach on turnover intentions through authenticity, belonging and justice. Consistent with the results of the main mediation model, the results showed that greater perceived supervisor identity consciousness predicted a higher sense of authenticity, belonging, and justice. Belonging and justice, but not authenticity, were in turn associated with lower turnover intentions. There were significant indirect effects of organizational diversity approach on organizational attractiveness through belonging and justice. None of the pairwise contrasts between the indirect effects were found to be significant.

We re-ran the mediation model twice and added knowledge of supervisor’s diversity approach and then organizational diversity approach as covariates. The pattern of results remained the same.

***Collapsing Authenticity and Belonging in the Model***

Lastly, we collapsed authenticity and belonging into a single variable of inclusion and explored its role in our model. We re-ran our mediation analysis from Study 3 with inclusion and justice as mediators. The results showed that greater perceived identity consciousness predicted a higher sense of inclusion (*b=*0.64 *SE=*0.04, *p*<.001, 95%CI[0.55, 0.72]). Inclusion was also a significant predictor of turnover intentions (*b=-*0.40 *SE=*0.09, *p*<.001, 95%CI[-0.57, -0.23]). The indirect effect through inclusion was significant (*b_indirect=_*-0.26, *SE*=0.06, 95%CI[-0.39,-0.14]). The pairwise contrast between inclusion and justice was not significant.

***Moderation by Gender***

We conducted a series of linear regression analyses to examine the difference between female (coded a 1) vs non-female (coded as 0) participants on turnover intentions, authenticity, belonging and justice. We also tested for an interaction effect between organizational diversity approach and female identity. We found no main or interactive effects of race/ethnicity on turnover intentions, authenticity, belonging, or justice (*ps>*.067).

**Means Tables per Gender Identity and Sexual Orientation for Studies 1, 2 and 3**

**Table 1S**

*Means and standard deviations of organizational attractiveness for sexual orientation by gender identity in Study 1*

|  |  | |  | Sexuality | | | | | | | | | | | | | | | | |
| --- | --- | --- | --- | --- | --- | --- | --- | --- | --- | --- | --- | --- | --- | --- | --- | --- | --- | --- | --- | --- |
|  |  | |  | Gay | | Lesbian | | Bisexual | | Pansexual | | Asexual | | Queer | | Hetero | | Total | | |
| Trans Identity | | |  | Blind | Consc | Blind | Consc | Blind | Consc | Blind | Consc | Blind | Consc | Blind | Consc | Blind | Consc | Blind | Consc |  |
| Yes | |  |  |  |  |  |  |  |  |  |  |  |  |  |  |  |  |  |  |  |
|  | Male | | *M*  *SD*  *n* | 4.80  *(-)*  *1* | *-*  *(-)*  *0* | *-*  *(-)*  *0* | *-*  *(-)*  *0* | 1.20  *(-)*  *1* | 4.30  *(0.42)*  *2* | *-*  *(-)*  *0* | 6.20  *(-)*  *1* | 5.50  *(1.55)*  *2* | *-*  *(-)*  *0* | *-*  *(-)*  *0* | 7  *(-)*  *1* | *-*  *(-)*  *-* | 7  *(-)*  *1* | 4.25  *(2.24)*  *4* | 5.60  *(1.78)*  *9* |  |
|  | Female | | *M*  *SD*  *n* | *-*  *(-)*  *0* | *-*  *(-)*  *0* | 2.30  *(1.55)*  *2* | *-*  *(-)*  *0* | 4.67  *(1.51)*  *3* | *-*  *(-)*  *0* | *-*  *(-)*  *0* | 5  *(0.29)*  *2* | 3.10  *(2.40)*  *2* | *-*  *(-)*  *0* | *-*  *(-)*  *0* | 6  *(-)*  *1* | *-*  *(-)*  *0* | *-*  *(-)*  - | 3.54  *(1.83)*  *7* | 5.33  *(0.61)*  *3* |  |
|  | Non-binary | | *M*  *SD*  *n* | *-*  *(-)*  *0* | *-*  *(-)*  *0* | *-*  *(-)*  *0* | *-*  *(-)*  *0* | 3.75  *(1.26)*  9 | 5.93  *(1.02)*  *3* | *4.60*  *(0.28)*  *2* | 5.78  *(0.46)*  *3* | 3.40  *(-)*  *1* | 5.62  *(1.48)*  *8* | 3.10  *(1.83)*  *2* | 5  *(-)*  *1* | *-*  *(-)*  0 | *-*  *(-)*  0 | 3.76  *(1.20)*  *14* | 5.66  *(1.15)*  *15* |  |
|  | Gender-fluid | | *M*  *SD*  *n* | -  *(-)*  0 | *-*  *(-)*  *0* | 4.50  *(0.42)*  *2* | *-*  *(-)*  *0* | 2.40  *(-)*  *1* | *-*  *(-)*  *0* | 5.20  *(-)*  *1* | *-*  *(-)*  *0* | *-*  *(-)*  *0* | *-*  *(-)*  *0* | *-*  *(-)*  *-* | 7  *(-)*  *1* | *-*  *(-)*  *0* | *-*  *(-)*  *0* | 4.15  *(1.23)*  *4* | 7  *(-)*  *1* |  |
|  | Agender | | *M*  *SD*  *n* | *-*  *(-)*  *0* | *-*  *(-)*  *0* | *-*  *(-)*  *0* | *-*  *(-)*  *0* | *-*  *(-)*  *0* | *-*  *(-)*  *0* | *-*  *(-)*  *0* | *-*  *(-)*  *0* | *-*  *(-)*  *0* | *-*  *(-)*  *0* | *-*  *(-)*  *0* | *-*  *(-)*  *0* | *-*  *(-)*  *0* | *-*  *(-)*  *0* | *-*  *(-)*  *0* | *-*  *(-)*  *0* |  |
|  | Self-Described | | *M*  *SD*  *n* | *-*  *(-)*  *0* | *-*  *(-)*  *0* | 4.40  *(-)*  *1* | *-*  *(-)*  *0* | 2  *(1.41)*  *2* | *5.20*  *(-)*  *1* | *-*  *(-)*  *0* | *-*  *(-)*  *0* | *-*  *(-)*  *0* | *-*  *(-)*  *0* | *-*  *(-)*  *0* | *-*  *(-)*  *0* | *-*  *(-)*  *0* | *-*  *(-)*  *0* | 3.10  *(2.10)*  *3* | 5.20  *(-)*  *1* |  |
|  |  | |  |  |  |  |  |  |  |  |  |  |  |  |  |  |  |  |  |  |
| No |  | |  |  |  |  |  |  |  |  |  |  |  |  |  |  |  |  |  |  |
|  | Male | | *M*  *SD*  *n* | 3.90  *(1.88)*  *29* | 5.10  *(1.66)*  *28* | *-*  *(-)*  *0* | *-*  *(-)*  *0* | 3.93  *(1.55)*  *16* | 5.17  *(0.99)*  *15* | 6  *(0.84)*  *2* | 4.20  *(-)*  *1* | 4.06  *(1.28)*  *3* | *-*  *(-)*  *0* | 5  *(-)*  *1* | *-*  *(-)*  *0* | *-*  *(-)*  *0* | *-*  *(-)*  *0* | 4.02  *(1.72)*  *51* | 5.10  *(1.44)*  *44* |  |
|  | Female | | *M*  *SD*  *n* | *-*  *(-)*  *0* | *-*  *(-)*  *0* | 4.21  *(1.74)*  *31* | 5.58  *(1.16)*  *24* | 3.60  *(1.54)*  *71* | 5.42  *(1.34)*  *80* | 2.44  *(1.72)*  *5* | 5.80  *(0.73)*  *9* | 3.94  *(1.50)*  *11* | 5.26  *(0.62)*  *7* | 3.38  *(1.52)*  *9* | 4.72  *(1.90)*  *8* | *-*  *(-)*  *0* | *-*  *(-)*  *0* | 3.72  *(1.61)*  *127* | 5.42  *(1.29)*  *128* |  |

**Table 2S**

*Means and standard deviations of organizational attractiveness for sexual orientation by gender identity in Study 2*

|  |  | |  | Sexuality | | | | | | | | | | | | | | | | |
| --- | --- | --- | --- | --- | --- | --- | --- | --- | --- | --- | --- | --- | --- | --- | --- | --- | --- | --- | --- | --- |
|  |  | |  | Gay | | Lesbian | | Bisexual | | Pansexual | | Asexual | | Queer | | Hetero | | Total | | |
| Trans Identity | | |  | Blind | Consc | Blind | Consc | Blind | Consc | Blind | Consc | Blind | Consc | Blind | Consc | Blind | Consc | Blind | Consc |  |
| Yes | |  |  |  |  |  |  |  |  |  |  |  |  |  |  |  |  |  |  |  |
|  | Male | | *M*  *SD*  *n* | 5  *(1.97)*  *2* | *-*  *(-)*  *0* | *-*  *(-)*  *0* | *-*  *(-)*  *0* | 4.32  *(1.37)*  *5* | 5.80  *(1.13)*  *2* | *-*  *(-)*  *0* | 5.60  *(0.85)*  *2* | 3.33  *(1.50)*  *3* | *-*  *(-)*  *0* | *-*  *(-)*  *0* | 5.67  *(0.41)*  *3* | 4.13  *(2.53)*  *3* | 5  *(-)*  *1* | 4.15  *(1.64)*  *13* | 5.6  *(0.63)*  *8* |  |
|  | Female | | *M*  *SD*  *n* | *-*  *(-)*  *0* | *-*  *(-)*  *0* | *-*  *(-)*  *0* | 5.07  *(2.84)*  *3* | 3.06  *(1.36)*  *3* | 4.40  *(1.21)*  *4* | *-*  *(-)*  *0* | 6.40  *(-)*  *1* | 4.2  *(-)*  *1* | 5  *(-)*  *1* | *-*  *(-)*  *0* | 5.60  *(1.98)*  *2* | *-*  *(-)*  *0* | *-*  *(-)*  *0* | 3.35  *(1.25)*  *4* | 5.04  *(1.69)*  *11* |  |
|  | Non-binary | | *M*  *SD*  *n* | 4.5  *(2.69)*  *2* | 4.6  *(-)*  *1* | 3.95  *(2.12)*  *4* | 5.87  *(0.61)*  *3* | 6  *(-)*  *1* | 4.42  *(1.85)*  *9* | 3.64  *(1.35)*  *13* | 5.80  *(0.42)*  *6* | 2.14  *(1.54)*  *7* | 5.25  *(0.72)*  *4* | 3.36  *(0.99)*  *5* | 5.24  *(1.93)*  *5* | *-*  *(-)*  *0* | *-*  *(-)*  *0* | 3.44  *(1.64)*  *32* | 5.14  *(1.42)*  *28* |  |
|  | Gender-fluid | | *M*  *SD*  *n* | *-*  *(-)*  *0* | *-*  *(-)*  *0* | *-*  *(-)*  *0* | *-*  *(-)*  *0* | 4.4  *(-)*  *1* | *-*  *(-)*  *0* | 4.9  *(1.55)*  *2* | 4.9  (0.42)  2 | *-*  *(-)*  *0* | *-*  *(-)*  *0* | 1.6  *(-)*  *1* | 4.8  *(0.56)*  *2* | *-*  *(-)*  *0* | *-*  *(-)*  *0* | 3.95  *(1.82)*  *4* | 4.85  *(0.41)*  *4* |  |
|  | Agender | | *M*  *SD*  *n* | *-*  *(-)*  *0* | *-*  *(-)*  *0* | *-*  *(-)*  *0* | *-*  *(-)*  *0* | *-*  *(-)*  *0* | *-*  *(-)*  *0* | *-*  *(-)*  *0* | *-*  *(-)*  *0* | *-*  *(-)*  *0* | *-*  *(-)*  *0* | *-*  *(-)*  *0* | *-*  *(-)*  *0* | *-*  *(-)*  *0* | *-*  *(-)*  *0* | *-*  *(-)*  *0* | *-*  *(-)*  *0* |  |
|  | Self-Described | | *M*  *SD*  *n* | *-*  *(-)*  *0* | *-*  *(-)*  *0* | 2  *(-)*  *1* | 5.20  *(1.40)*  *3* | 1.20  *(-)*  *1* | *-*  *(-)*  *0* | *-*  *(-)*  *0* | 3.6  *(-)*  *1* | 6.40  *(-)*  *1* | 7  *(-)*  *1* | *-*  *(-)*  *0* | *-*  *(-)*  *0* | *-*  *(-)*  *0* | *-*  *(-)*  *0* | 3.20  *(2.80)*  *3* | 5.24  *(1.56)*  *5* |  |
|  |  | |  |  |  |  |  |  |  |  |  |  |  |  |  |  |  |  |  |  |
| No |  | |  |  |  |  |  |  |  |  |  |  |  |  |  |  |  |  |  |  |
|  | Male | | *M*  *SD*  *n* | 4.80  *(1.29)*  *34* | 5.43  *(1.20)*  *26* | *-*  *(-)*  *0* | *-*  *(-)*  *0* | 4.39  *(1.65)*  *28* | 5.44  *(1.21)*  *26* | 3.72  *(2.22)*  *5* | 6.00  *(1.22)*  *3* | 4.95  *(2.68)*  *4* | 3.1  *(1.84)*  *2* | 3  *(0.56)*  *2* | *-*  *(-)*  *0* | *-*  *(-)*  *0* | *-*  *(-)*  *0* | 4.53  *(1.59)*  *73* | 5.38  *(1.27)*  *57* |  |
|  | Female | | *M*  *SD*  *n* | *-*  *(-)*  *0* | 5.20  *(-)*  *1* | 4.13  *(1.67)*  *17* | 5.81  *(0.74)*  *15* | 3.97  *(1.47)*  *59* | 5.43  *(0.97)*  *15* | 4.64  *(1.37)*  *9* | 5.60  *(1.12)*  *14* | 5.42  *(0.79)*  *8* | 5.87  *(0.88)*  *8* | 3.85  *(1.50)*  *11* | 5.28  *(1.73)*  *5* | *-*  *(-)*  *0* | *-*  *(-)*  *0* | 4.15  *(1.49)*  *104* | 5.52  *(0.99)*  *115* |  |

**Table 3S**

*Means and standard deviations of turnover intentions for sexual orientation by gender identity in Study 3*

|  |  |  |  |  | Sexuality |  |  |  |  |  | | |
| --- | --- | --- | --- | --- | --- | --- | --- | --- | --- | --- | --- | --- |
| Trans Identity |  |  | Gay | Lesbian | Bisexual | Pansexual | Asexual | Queer | Heterosexual | | Self-Described | Total |
| Yes |  |  |  |  |  |  |  |  |  | |  |  |
|  | Male | *M*  *SD*  *n* | 4.25  *(2.22)*  *4* | *-*  *(-)*  *0* | 4.78  *(1.93)*  *7* | 5.25  *(1.77)*  *2* | 4.67  *(0.73)*  *3* | *-*  *(-)*  *0* | 4.30  *(2.22)*  *5* | | *7*  *(-)*  *1* | 4.70  *(1.81)*  *22* |
|  | Female | *M*  *SD*  *n* | *-*  *(-)*  *0* | 4.25  *(2.46)*  *4* | 3.70  *(1.95)*  *5* | 6.33  *(1.15)*  *3* | 5.50  *(-)*  *1* | *-*  *(-)*  *0* | *-*  *(-)*  *0* | | *-*  *(-)*  *0* | 4.61  *(2.05)*  *13* |
|  | Non-binary | *M*  *SD*  *n* | 3.00  *(0.00)*  *2* | 5.25  *(1.99)*  *6* | 4.79  *(1.79)*  *17* | 3.37  *(2.61)*  *8* | 3.58  *(1.88)*  *6* | 4.79  *(2.03)*  *12* | *-*  *(-)*  *0* | | *-*  *(-)*  *0* | 4.41  *(2.03)*  *51* |
|  | Genderfluid | *M*  *SD*  *n* | *-*  *(-)*  *0* | 7  *(-)*  *1* | 2.75  *(1.77)*  *2* | 3.75  *(3.18)*  *2* | *-*  *(-)*  *0* | 7  *(-)*  *1* | *-*  *(-)*  *0* | | *-*  *(-)*  *0* | 4.50  *(2.57)*  *6* |
|  | Agender | *M*  *SD*  *n* | 5.00  *(-)*  *1* | 3.50  *(-)*  *1* | 4.00  *(-)*  *1* | *-*  *(-)*  *0* | *-*  *(-)*  *0* | 6.50  *(-)*  *1* | *-*  *(-)*  *0* | | *-*  *(-)*  *0* | 4.75  *(1.32)*  *4* |
|  | Self-Described | *M*  *SD*  *n* | *-*  *(-)*  *0* | *-*  *(-)*  *0* | *-*  *(-)*  *0* | 4.00  *(1.32)*  *3* | *-*  *(-)*  *0* | 1.75  *(0.35)*  *2* | *-*  *(-)*  *0* | | 1.00  *(-)*  *1* | 2.75  *(1.63)*  *6* |
|  |  |  |  |  |  |  |  |  |  | |  |  |
| No |  |  |  |  |  |  |  |  |  | |  |  |
|  | Male | *M*  *SD*  *n* | 3.96  *(2.03)*  *62* | *-*  *(-)*  *0* | 3.45  *(1.94)*  *45* | 2.50  *(2.02)*  *6* | 2.40  *(1.56)*  *5* | 4.00  *(-)*  *1* | *-*  *(-)*  *0* | | *-*  *(-)*  *0* | 3.63  *(2.00)*  *119* |
|  | Female | *M*  *SD*  *n* | 1.00  *(-)*  *1* | 3.28  *(2.06)*  *68* | 3.37  *(2.02)*  *118* | 4.64  *(1.91)*  *18* | 3.15  *(1.45)*  *10* | 3.28  *(2.14)*  *9* | *-*  *(-)*  *0* | | *-*  *(-)*  *0* | 3.42  *(2.02)*  *224* |

**Table 4S**

*Correlations between Items of the Organizational Diversity Approach Scale*

| Variable | 1 | 2 | 3 | 4 | 5 | 6 |
| --- | --- | --- | --- | --- | --- | --- |
| 1. My organization believes that employees’ demographic differences should be acknowledged and valued | - |  |  |  |  |  |
| 2. My organization behaves in ways that acknowledge employees’ demographic differences | .85^**^ | - |  |  |  |  |
| 3. My organization exposes its employees to other employees’ different demographic backgrounds | .62^**^ | .64^**^ | - |  |  |  |
| 4 My organization believes that acknowledging other employees’ demographic differences is the best way to approach diversity | .85^**^ | .84^**^ | .65^**^ | - |  |  |
| 5. My organization makes an effort to NOT expose its employees to other employees’ different demographic backgrounds | -.30^**^ | -.29^**^ | -.41^**^ | -.31^**^ | - |  |
| 6. My organization believes that NOT acknowledging others' demographic differences is the best way to approach diversity | -.58^**^ | -.56^**^ | -.44^**^ | -.59^**^ | .44^**^ |  |

*Note*. ^+^ *p*<.10, ^*^*p*<.05, ^**^*p*<.01

**Figure 1S**

***Interaction Effect Between Diversity Approach and Tenure on Turnover Intentions***


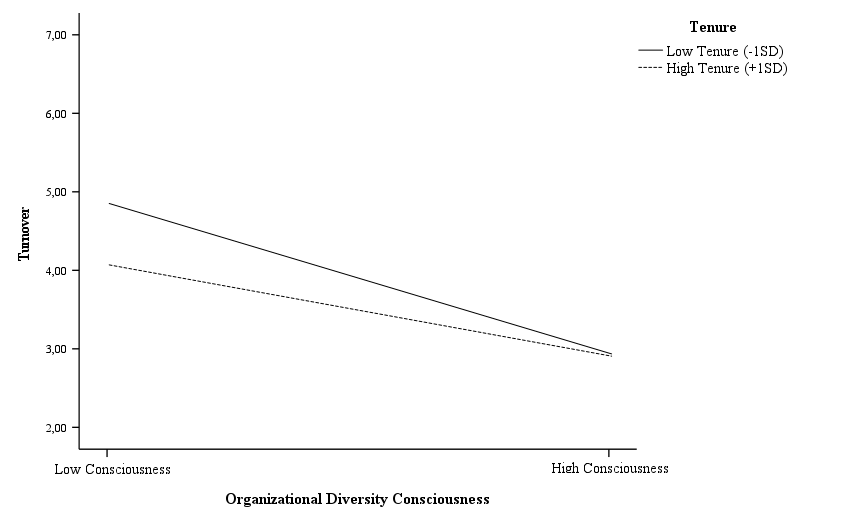


**Figure 2S**

***Interaction Effect Between Diversity Approach and Tenure on Authenticity***


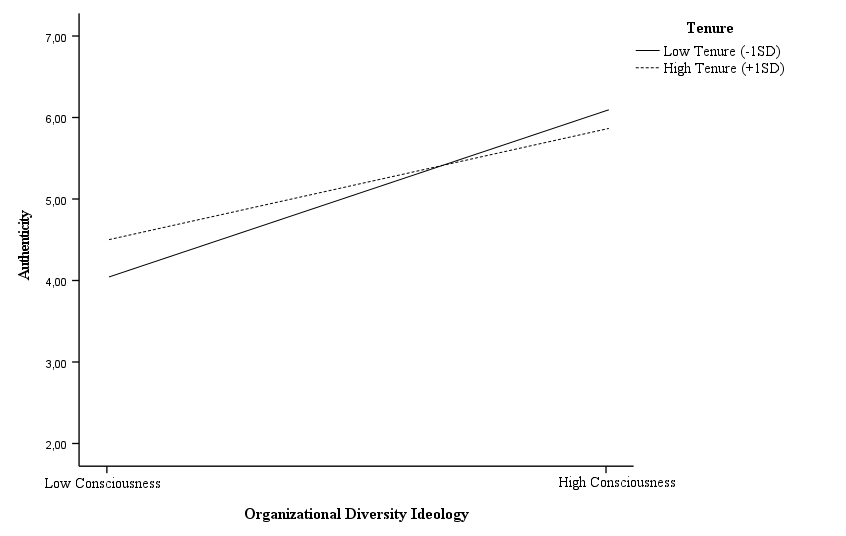


**Figure 3S**

***Interaction Effect Between Diversity Approach and Tenure on Belonging***


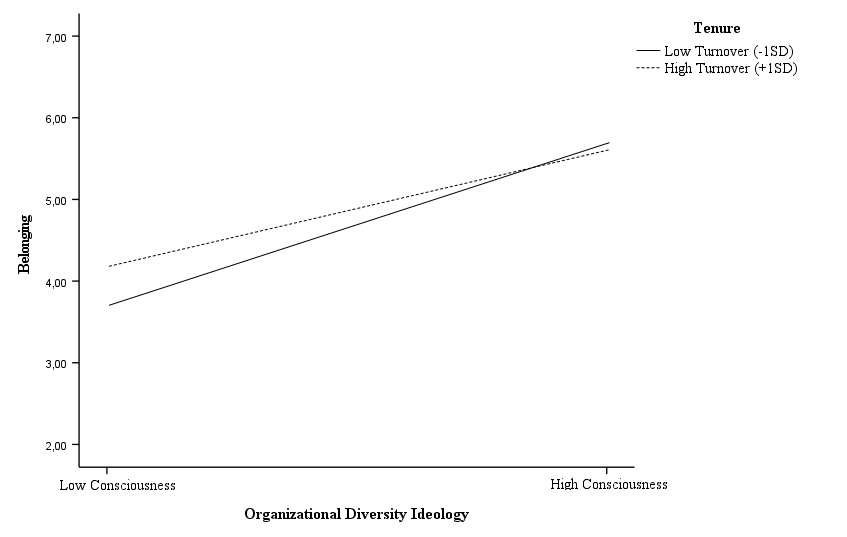


**References**

Conway, J. M., & Huffcutt, A. I. (2003). A review and evaluation of exploratory factor analysis practices in organizational research. *Organizational Research Methods*, *6*(2), 147–168. https://doi.org/10.1177/1094428103251541

Cvetkovska, S., Verkuyten, M., Adelman, L., & Yogeeswaran, K. (2021). Being tolerated: Implications for well-being among ethnic minorities. *British Journal of Psychology*, *112*(3), 781–803. https://doi.org/10.1111/bjop.12492

Dang, C. T., Volpone, S. D., & Umphress, E. E. (2022). The ethics of diversity ideology: Consequences of leader diversity ideology on ethical leadership perception and organizational citizenship behavior. *Journal of Applied Psychology, 108(2),* 307–329. https://doi.org/10.1037/apl0001010

Hahn, A., Banchefsky, S., Park, B., & Judd, C. M. (2015). Measuring intergroup ideologies: Positive and negative aspects of emphasizing versus looking beyond group differences. *Personality and Social Psychology Bulletin*, *41*(12), 1646–1664. https://doi.org/10.1177/0146167215607351

Hayes, A. F. (2017). *Introduction to mediation, moderation, and conditional process analysis, Second edition: A regression-based approach*. Guilford Publications. http://ebookcentral.proquest.com/lib/uunl/detail.action?docID=5109647

Mohr, J., & Fassinger, R. (2000). Measuring dimensions of lesbian and gay male experience. *Measurement and Evaluation in Counseling and Development*, *33*(2), 66–90. https://doi.org/10.1080/07481756.2000.12068999

Simmering, M. J., Fuller, C. M., Richardson, H. A., Ocal, Y., & Atinc, G. M. (2015). Marker variable choice, reporting, and interpretation in the detection of common method variance: A review and demonstration. *Organizational Research Methods*, *18*(3), 473–511. https://doi.org/10.1177/1094428114560023

1. As detailed in the preregistration, Study 2 and 3 included a single item tolerance measure. However, we do not discuss this measure in detail here because we plan to explore it more deeply in a separate study. [↑](#footnote-ref-1)
